# Histomorphological and morphometric characterization of Nile tilapia (*Oreochromis niloticus*) ovaries, with emphasis on processes and structures related to the ovarian regressing phase

**DOI:** 10.1007/s10695-026-01740-x

**Published:** 2026-07-10

**Authors:** Isabela Machado de Oliveira, Salmo Azambuja de Oliveira, Monica Cassel

**Affiliations:** 1 Departamento de Biociências e Saúde (DBS), Instituto de Ciências Agrárias, Exatas e Biológicas de Iturama, Universidade Federal do Triângulo Mineiro, Campus Iturama (ICAEBI-UFTM), Iturama, MG Brazil; 2 Departamento de Biologia Estrutural, Instituto de Ciências Biomédicas, Universidade Federal de Alfenas (UNIFAL), Alfenas, MG Brazil

**Keywords:** First protocol, Fish farming, Histomorphological modifications, Involutional processes, Oogenesis, Reproduction

## Abstract

Understanding fish reproductive characteristics, specifically ovarian development and regression, is essential for optimizing the performance of commercially important species like Nile tilapia (*Oreochromis niloticus*). This study characterizes the histomorphological modifications associated with follicular atresia and post-ovulatory complexes (POCs) during the ovarian regressing phase. Genetically improved (GIFT-type) female fish were monitored over a 60-day reproductive management period, with sequential sampling (G0–G7, *n* = 20) performed before and after de-mating. Ovaries were evaluated via gonadosomatic index (GSI) and detailed histological analysis. Macroscopically, three ovarian patterns were identified: “final maturation,” “post-spawning,” and a previously undescribed “hardened” pattern. Significant differences in ovarian weight and GSI supported a multiple-batch spawning strategy, while histology confirmed asynchronous folliculogenesis. During regression, early-stage atresia and POCs predominated up to 21 days post-unmating. By days 28–35, ovarian functional recovery was assessed by observing later atretic stages and increased mature follicles. These findings reveal that ovarian remodeling occurs gradually through tissue reabsorption. Based on these data, we propose an evidence-based 60-day resting protocol for broodstock reintroduction to maximize gonadal recovery, reproductive performance, and sustainability in aquaculture systems.

**Graphical Abstract:**

**Figure:**
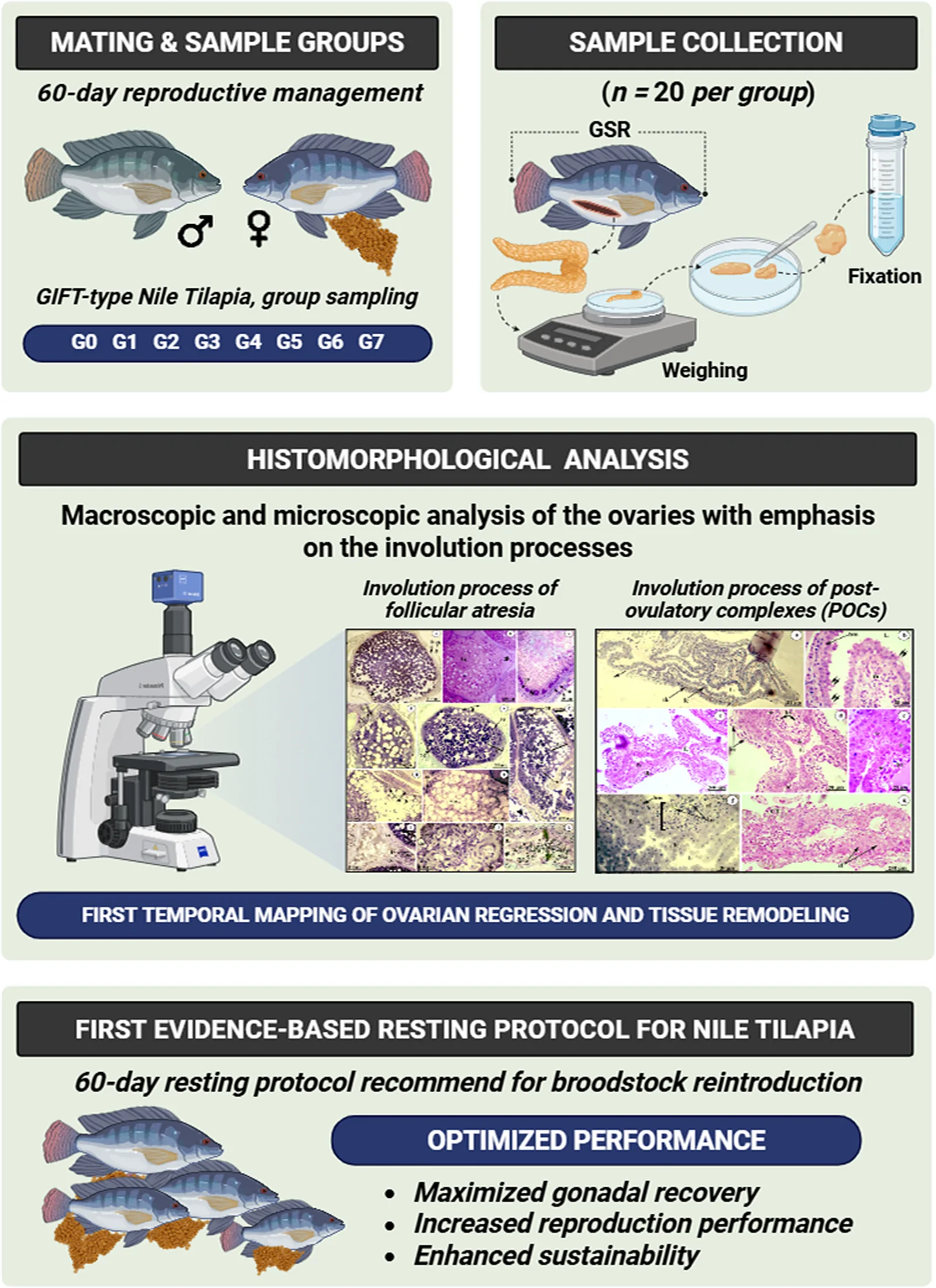

**Supplementary Information:**

The online version contains supplementary material available at https://doi.org/10.1007/s10695-026-01740-x.

## Introduction

Knowledge on the reproductive characteristics of fish is essential for understanding the adaptations developed by each species to enhance reproductive success in a given environment, considering life history aspects (Mazzoni et al. 2005; Cassel 2019). The physiological processes involved in fish reproduction mainly include gonad differentiation, gametogenesis, gamete release, fertilization, and egg hatching (Ribeiro and Moreira 2018). For this reason, oocyte growth and development, along with the proportion and distribution of developmental stages (folliculogenesis, primary or pre-vitellogenic growth, secondary or vitellogenic growth, and maturation), are important processes in the reproductive biology of native and farmed fish (Tyler and Sumpter 1996; Lubzens et al. 2010; Mai and Lemos 2021).

When evaluating the reproductive stage in female fish, description and quantification of ovarian structures can be used as a guide to classify ovaries into different reproductive phases: developing, spawning-capable, regressing, and regenerating (Brown-Peterson et al. 2011). As described by Brown-Peterson et al. (2011), during the developing phase the female prepares for a new reproductive cycle: oocyte preparation begins, increasing cell volume and undergoing cell differentiation. In this phase, a greater presence of primary oocytes is observed, mainly in the cortical alveoli stage, as well as secondary or vitellogenic oocytes. Next, during the spawning-capable phase, the oocytes pass through the final stages of maturation, followed by the spawning process (Brown-Peterson et al. 2011). In the regressing phase, the focus of this study, the presence of atretic follicles and post-ovulatory complexes (POCs) can be observed. Individuals remain in this phase for a relatively short time and then move on to the regenerating phase, an intermediate phase between regressing and ovarian development, when the fish prepare for the next reproductive cycle (Brown-Peterson et al. 2011).

The regressing phase encompasses the period after spawning, namely, the end of the reproductive process and preparation for a new reproductive cycle. This phase is characterized by the presence of follicular atresia, POCs, and few (if any) healthy vitellogenic oocytes (Quagio-Grassiotto et al. 2013; Cassel et al. 2017a). The presence of degenerative structures such as atretic follicles and POCs is important histological indicators of reproductive dynamics in fish. These degenerative structures undergo processes of involution, with the reabsorption of structures that will no longer be used, an important phenomenon with a significant impact on tissue remodeling for ovarian function (Wildner et al. 2013; Melo et al. 2014).

Among these involutional processes, atresia is a term used to describe the degradation and reabsorption of gametes; follicular atresia is a degenerative process by which the ovarian follicles of vertebrates lose their integrity and are eliminated (Kennedy 2002; Santos et al. 2008; Thomé et al. 2009). The process of oocyte atresia can occur throughout the reproductive cycle, but is most commonly observed in maturing and mature oocytes, coinciding with the pre-spawning and post-spawning phases (Guraya 1994; Mai and Lemos 2021). Furthermore, an increase in the occurrence of follicular atresia above physiological rates reduces fish fecundity and can even cause reproductive failures in wild and farmed fish stocks. This phenomenon consequently has a wide range of implications in applied sciences, such as fishing and aquaculture (Corriero et al. 2021; Qiang et al. 2022).

In teleosts, vitellogenic oocytes that were not ovulated during the reproductive period become atretic and are slowly reabsorbed during post-spawning ovarian regression (Rizzo and Bazzoli 1995; Miranda et al. 1999; Santos et al. 2005; Morais et al. 2012; Melo et al. 2014). Since teleost oocytes contain a large amount of yolk and are produced in large numbers, phagocytosis by granulosa cells appears to be involved in the process of recycling this valuable energy reserve produced by females (Linares-Casenave et al. 2002; Eykelbosh and Van der Kraak 2010). During the atresia process, the degenerating follicles are phagocytosed by somatic cells (mainly follicular and theca cells), favoring reuse of nutrients by the fish (Vazzoler 1996; Grier et al. 2009).

Other structures may also be reabsorbed after the spawning period, such as POCs, which are remnants of ovarian follicles composed of follicular cells, a basement membrane, and theca cells (Grier et al. 2007). After spawning, a newly formed POC remains attached to the germinal epithelium (Grier 2012; Melo et al. 2014). In oviparous fish, these structures do not exhibit hormonal activity (unlike the corpus luteum in mammals) and are transient structures after spawning (Selman and Wallace 1989; Drummond et al. 2000; Santos et al. 2005). For this reason, the removal of POCs may be essential for the recruitment of new follicles for the next reproductive season (Kumar and Joy 2015).

Events such as follicular atresia and reabsorption of POCs are consequently fundamental for regulation of ovarian function in teleosts, promoting the removal of unfertilized or non-viable structures and preparing ovarian tissue for new follicular development cycles (Wallace and Selman 1990; Pandit et al. 2015). These structures that remain from the spawning process also serve as excellent experimental models for studying mechanisms of programmed cell death and autophagy, especially due to tissue remodeling involving follicular growth and regression (Santos et al. 2005, 2008; Thomé et al. 2006, 2012; Morais et al. 2012; Melo et al. 2014; Cassel et al. 2017b). Furthermore, despite the recognized importance of follicular atresia and POC resorption for ovarian remodeling, the regressing phase remains one of the least elucidated stages of the reproductive cycle. Detailed histomorphological and morphometric descriptions are still scarce, particularly along a temporal scale of recovery, limiting both biological understanding of ovarian dynamics and optimization of reproductive management in aquaculture species. And little is known about the structures that characterize the regressing phase (see Melo et al. 2014), especially when considering involutional processes along a temporal scale of gonadal recovery.

The study of ovarian structures related to the regressing period is consequently very important, especially in species of commercial interest, since the processes of gonadal recovery for new reproductive cycles can impact productivity and fecundity. To guarantee desired reproductive rates, with sufficient spawning frequency, eggs of adequate size, good semen quality, and good fertilization and hatching rates, broodstock and breeders must periodically undergo a process known as reproductive rest or resting (SENAR 2017).

The animal model for this study was the Nile tilapia (*Oreochromis niloticus*), a species that mainly stands out for its widespread cultivation in tropical and subtropical countries; it has gained popularity in recent years in Brazil for motives including rapid growth, hardiness, acceptance of balanced feeds, and excellent quality meat (Antonio 2006). Tilapia reproduce naturally throughout the year when conditions are suitable. Nile tilapia present a multiple-batch spawning pattern, with spawning occurring 8 to 12 times a year (Oliveira et al. 2007; SENAR 2017). The significant economic interest in this species has driven several reproductive studies on topics such as ovarian characterization (Bezerra 2024), testicular characterization (Schulter and Vieira Filho 2017), reproductive cycle analysis (Albuquerque 2022), fecundity analysis, and economic viability (Schulter and Vieira Filho 2017). However, a constant search for the ideal balance between price and production requires information related not only to practical aspects and technologies directed at the production of juveniles, but also ovarian physiology and recovery aspects (Almeida 2013).

For these reasons, the overarching objective of this study was to describe the ovarian morphology of Nile tilapia based on anatomical, histological, and morphometric characteristics, delving into details not yet considered in the literature. We also attempted to provide more robust and detailed data on ovarian histomorphological and morphometric characteristics with a focus on the regressing process in fish, especially in commercially significant species such as Nile tilapia. An additional goal was to understand the timescale necessary to establish a new reproductive cycle for the species without compromising production efficiency, improving biotechnological development and cultivation techniques applied to reproduction in teleost fish.

## Materials and methods

### Specimen maintenance and reproductive management

**Fig. 1:**
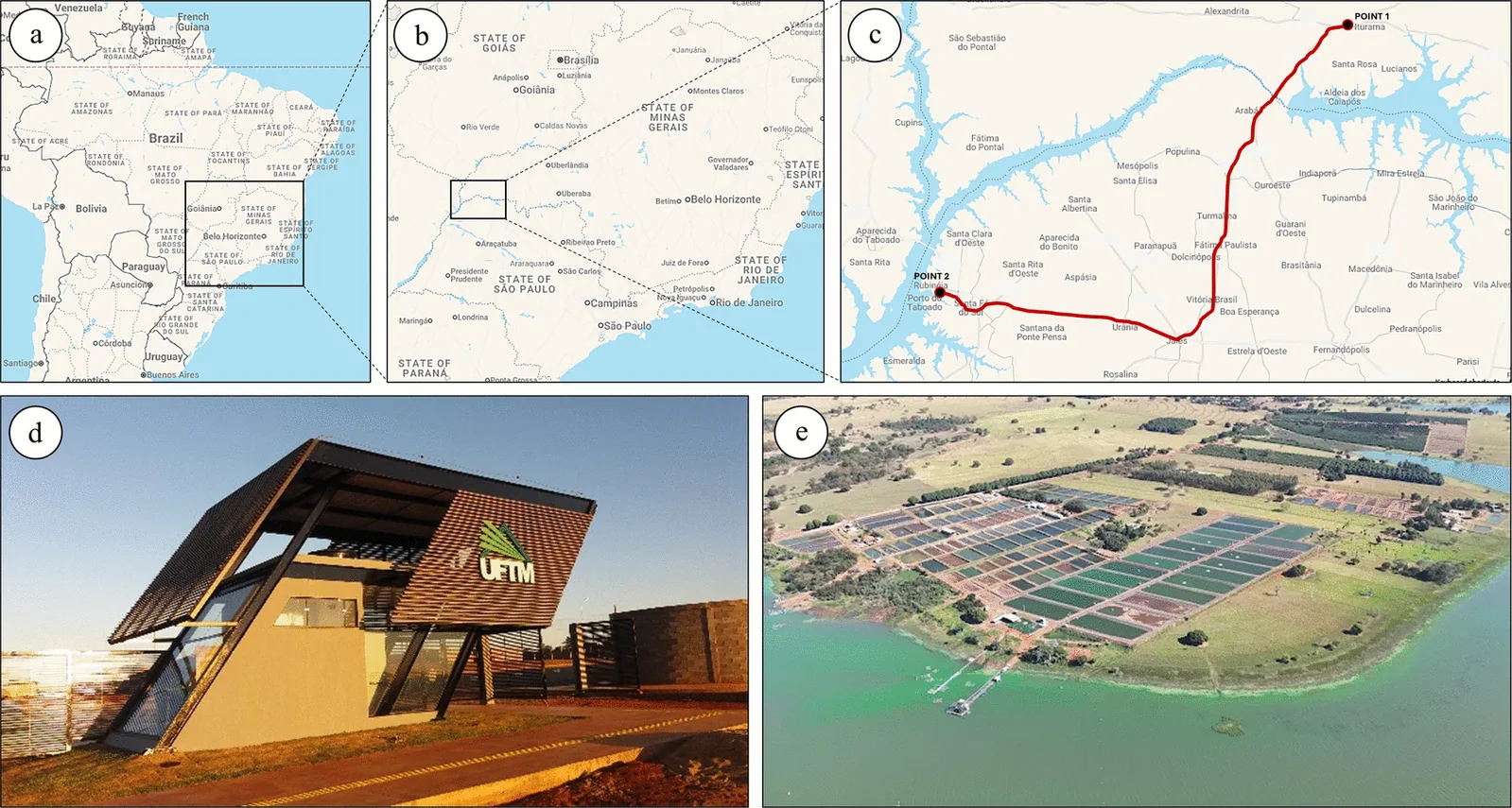
Map of the sampling site. **a** Southeast region of Brazil (square). **b** Zoom detail of the Triângulo Mineiro region (square) in the state of Minas Gerais. **c** Route between the Universidade Federal do Triângulo Mineiro (UFTM, point 1), and the Global Fish Aquaculture fish farm (point 2). **d** Facade of UFTM in Iturama, Minas Gerais. **e** Aerial view of the Global Fish Aquaculture fish farm located in Rubineia, São Paulo

Two-year-old female GIFT (genetically improved farmed tilapia)-type Nile tilapia (UEM/Codapar) from the Global Peixe Aquicultura facilities in Rubinéia, São Paulo, Brazil (20° 18′ 44″ S 51° 2′ 7″ W; Fig. 1) were selected for this study. GIFT-type females exhibit fast growth and superior growth in aquaculture systems while maintaining high fertility (Rossato et al. 2022). The fish were transitioned into their second reproductive period in the fish farm setting. A resting period was observed between the first and second reproductive periods during the winter, when the specimens were separated and kept apart in 3 × 3 m net cages containing approximately 2000 fish per cage (weighing 450 to 500 g each). The cages, located in a river branch, had a constantly renewed natural water source. The specimens were subject to a natural photoperiod (i.e., they are exposed to the natural, unmodified daily light and dark cycles corresponding to the time of year and the geographical latitude of the study location), separated by sex, and hand-fed twice daily on herbivorous feed (36% vegetable protein). From this resting group, 25 non-spawning females with ovaries full of eggs were collected to serve as the control group (G0) to verify the end of the ovarian regressing phase.

Due to higher temperatures (~ 28 °C) in the region at the end of July 2025, individuals that were previously separated by sex were paired and placed in the same pond (still within the fish farm) at a ratio of 2.5 females to each male. The breeding ponds are excavated, measuring roughly 20 × 50 m (1.5 m deep), and hold approximately 2600 animals per pond. The ponds have a constantly renewed natural water source (pumped from the river), and the fish were subject to a natural photoperiod and fed carnivorous feed (40% animal protein) once a day, every other day.

Reproductive management was maintained continuously for approximately 60 days. The sample size was 20 females per group, with the following sample groups established after reproductive management:

- Group 1 (G1): after the reproductive management period, on the same day as unmating.
- Group 2 (G2): females 3 days after unmating.
- Group 3 (G3): females 7 days after unmating.
- Group 4 (G4): females 14 days after unmating.
- Group 5 (G5): females 21 days after unmating.
- Group 6 (G6): females 28 days after unmating.
- Group 7 (G7): females 35 days after unmating.

### Collection of specimens and macroscopic analysis of the ovaries

After the females were separated into the sampling groups, and prior to any dissection procedure, all animals were euthanized via anesthetic overdose (3% eugenol/alcohol solution per 100 g of fish, according to Aoki and Moraes 2007) until opercular beating ceased, in accordance with the procedure approved by our institutional ethics review board for animal experimentation (CEUA/UFTM process 23,755.000337/2025–56). The specimens were photographed and measured according to standard length values in centimeters and total weight in grams. Both ovaries were then dissected, photographed, measured and weighed separately, and macroscopically characterized according to criteria such as color, structures present, weight, length and width, and variations of these factors in the sampled groups.

We also calculated the gonadosomatic index (GSI) for all females, defined as the ovarian weight as a percentage of total body weight, according to the equation by Vazzoler (1996): GSI = (Po Pt⁻^1^) × 100. In this equation, Po represents the combined total weight of both ovaries (since the ovaries were weighed separately) and Pt represents the total weight of the specimen. Quantitative data (weight, length, and width) and variation in GSI were compared between the sampled groups to assist in characterization of the ovary and modifications related to the ovarian regressing phase in this species.

Immediately after macroscopic observation, the ovaries were fixed in Karnovsky’s solution (2% glutaraldehyde and 4% paraformaldehyde in Sorensen buffer [0.1 M at pH 7.2]) or 4% paraformaldehyde (buffered with sodium phosphate solution [0.1 M at pH 7.4]) for 24 h. All steps, from anesthesia, euthanasia, and specimen dissection to immersing the gonads in the fixatives, were performed in the fish farm facilities. Following collection, the ovaries were transported to our university laboratory for further procedures. After the stipulated fixation time, the ovaries were removed from the fixatives and stored in 70% ethanol.

### Histomorphological and morphometric analyses of the ovaries

Ovaries from five females in group 0 were fixed in 4% paraformaldehyde and processed whole to verify the distribution of ovarian structures throughout the organ, since some fish species have regionalization in their ovaries and consequent asymmetrical distribution of structures. These ovaries were then transferred to histological processing cassettes, dehydrated in increasing concentrations of ethanol, cleared in xylene, and embedded in paraplast. The blocks were sectioned semi-serially to 5 μm thickness, and the sections were placed on slides pre-prepared with adhesive (egg white + glycerin) and left in a 50 °C oven overnight to dry and ensure fixation to the slide. Sections underwent periodic acid-Schiff’s reactive (PAS), ferric hematoxylin, and metanil yellow staining (Quintero-Hunter et al. 1991), and were then dehydrated overnight in a 60 °C oven and mounted with a coverslip and Erv-Mount (EasyPath) mounting medium. Finally, the slides were examined and photo-documented using a TNB-40 T-PL light microscope (OPTON, China) coupled with a Canon EOS R-100 digital camera (Canon Inc., Japan).

In the analysis of the ovaries, a homogeneous and symmetrical distribution of ovarian structures could be observed. We consequently opted to utilize random similar-sized fragments of these organs for the remaining histomorphological evaluation.

In this process, ovaries fixed in Karnovsky’s solution were fragmented; some of the fragments were dehydrated with increasing concentrations of ethanol, infiltrated with a 100% ethanol solution added to glycol methacrylate plastic resin (Historesin, Leica) in a 1:1 ratio for 2 h, and then infiltrated for another week at 4 °C with a pure resin solution. Finally, the fragments were embedded in the same resin with added catalyst and placed in plastic molds. The resin blocks were produced and cut semi-serially into 4 µm thick sections with approximately 1000 µm between each cut in order to avoid observing the same structures between sections (Duponchelle et al. 2000). The sections were placed on slides pre-prepared with adhesive (egg white + glycerin) and left to dry in a 50 °C oven overnight. Some sections were stained with PAS + ferric hematoxylin + metanil yellow, as described previously, while others were stained with hematoxylin and eosin (HE), following the standard staining protocol. Finally, the stained slides were mounted and analyzed as described earlier.

Analysis of microscopic characteristics and identification/description of germline and somatic cell types and their respective characteristics were based on staining affinities, as proposed by Grier (2012) and Cassel et al. (2017a, b). The descriptions considered the general appearance of the structures in order to take into account oocyte development and involutional processes of the species, as well as the standard characteristics present in all groups. Morphometric analyses were performed to compare the sampled groups.

Based on these characteristics, we were able to establish stages for the involution process of follicular atresia and POCs, and to count the stages in the involution processes. The involution stages of follicular atresia and POCs were determined by counting all stages of these structures present in three histological sections per individual, for all individuals and all sample groups. These data were then used to verify the progression of the ovarian regression process according to subsequent statistical analyses.

### Statistical analyses

All quantitative results were tested for normal distribution using the Lilliefors test. The test result was *p* < 0.001, indicating that none of the data presented a normal distribution; as a result, the non-parametric Kruskal–Wallis test and paired Wilcoxon test were used for each treatment. These tests were performed using the Systat 10 program, and the significance level was set at *p* ≤ 0.05.

## Results and discussion

### Macroscopic characteristics of the ovaries

**Fig. 2:**
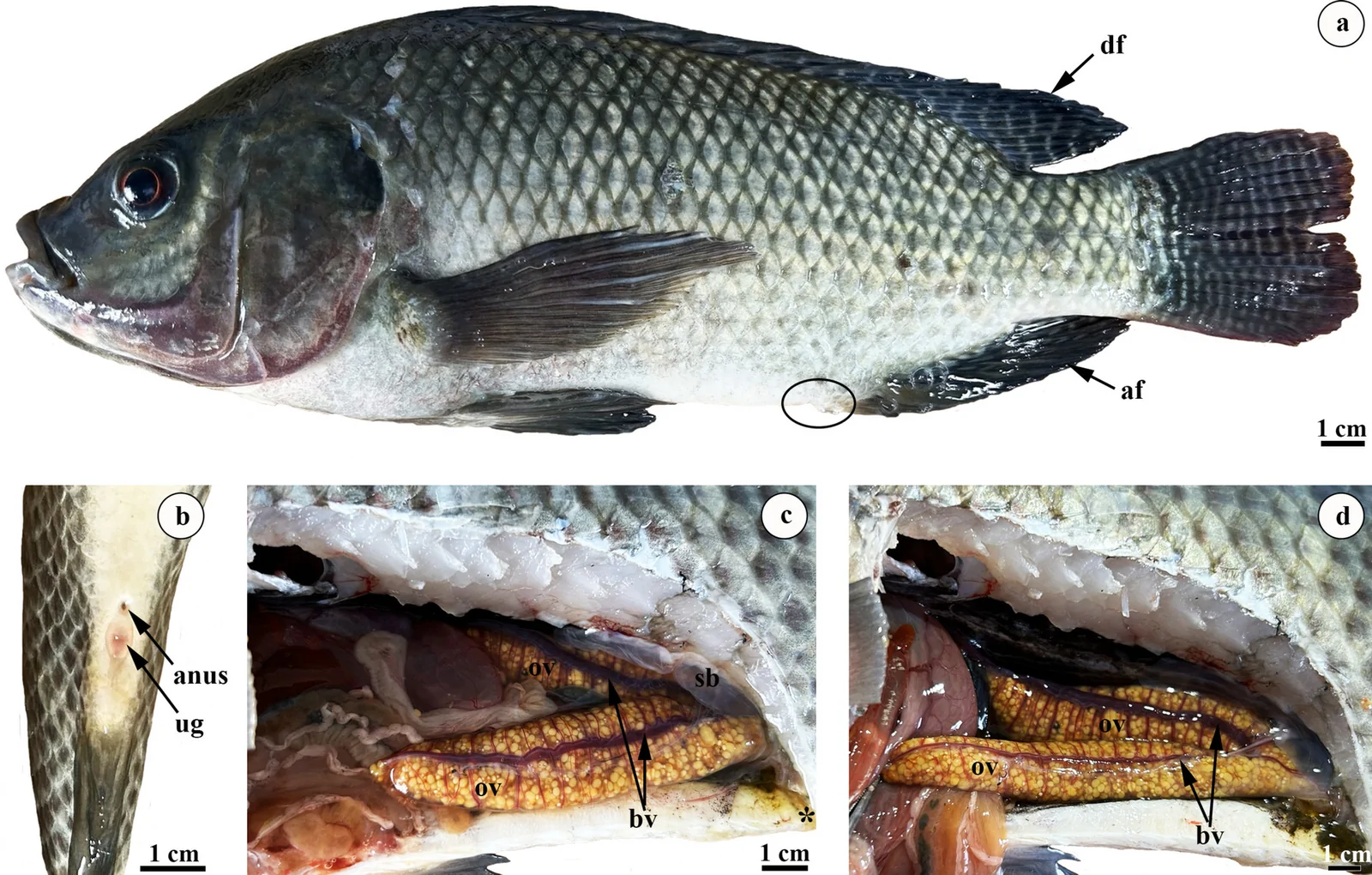
Female Nile tilapia (*O. niloticus*) specimens collected during the study. **a**, **b** Females are grayish in color, with rounded dorsal (df) and anal (af) fins and a divided and rounded urogenital opening (ug, circled in the photo). **c**, **d** The ovaries (ov) are paired, elongated, and vascularized organs (bv) and can be observed **c** in the coelomic cavity, **d** positioned below the swim bladder (sb) and close to the urogenital opening (*) once the organs were retracted

Nile tilapia females are grayish in color (Fig. 2a), with rounded dorsal and anal fins and a divided and rounded urogenital opening (Fig. 2b). The ovaries are located in the coelomic cavity, positioned below the swim bladder and close to the urogenital opening, through which the oocytes are released during spawning (Fig. 2c, d). They are paired, elongated, and vascularized organs, a pattern we observed in all the individuals (Fig. 2c, d). These characteristics are also in accordance with the classic anatomical descriptions for teleost fish in general, and specifically for Nile tilapia (El-Sayed 2019).

**Fig. 3:**
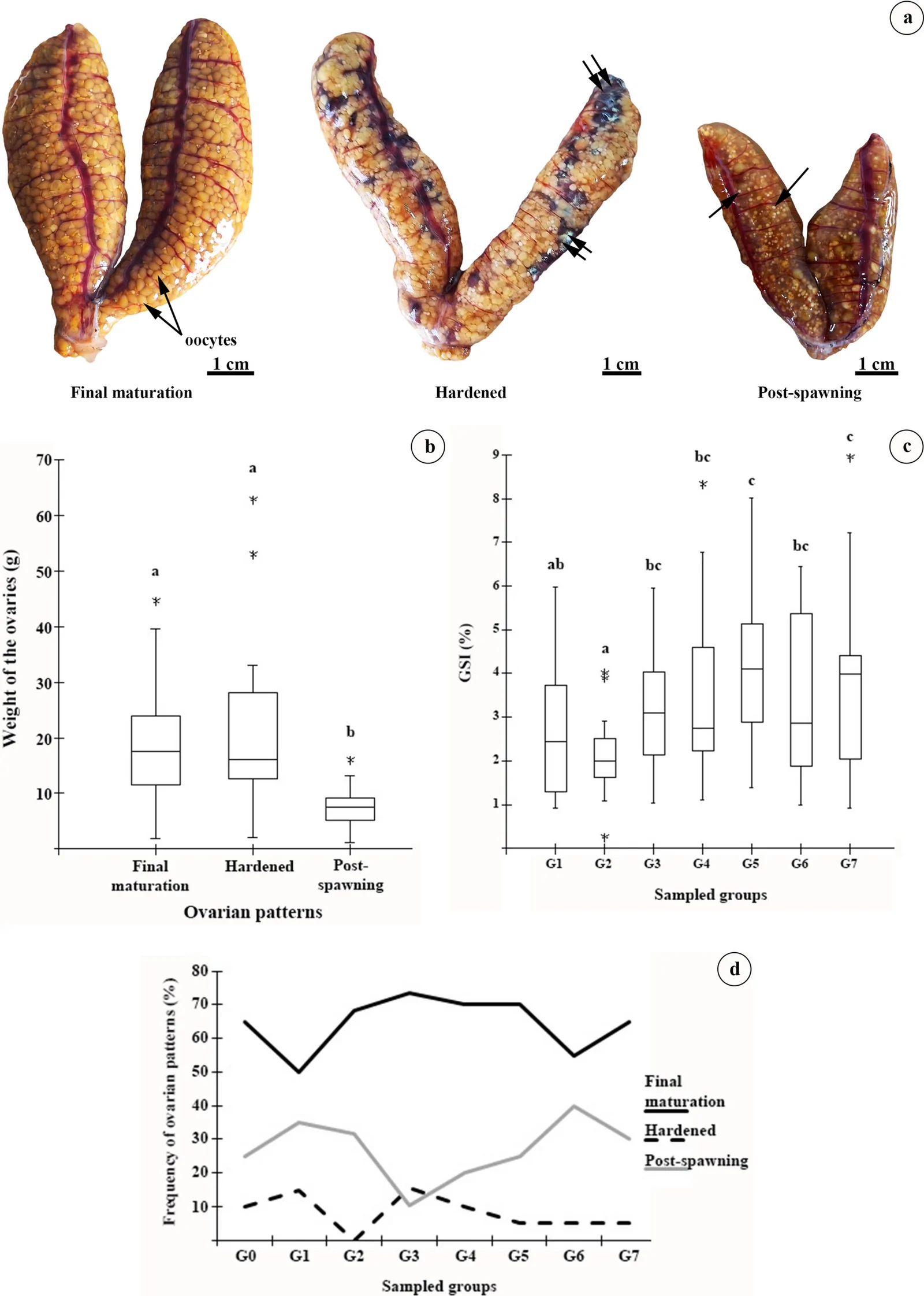
Macromorphometric characteristics of tilapia ovaries. **a** Ovaries were classified into three patterns: “final maturation,” with intense yellowish coloration and large oocytes; “hardened,” with intense yellow coloration and purplish/blackened areas (double arrows); and “post-spawning,” translucent red in color, with smaller and whitish oocytes (arrows). **b** Weight differed significantly between patterns, especially in the “post-spawning” group. **c** Gonadosomatic index (GSI) also varied significantly between groups, especially groups G1 and G2 compared to the other groups. **d** Ovaries classified as “final maturation” were most frequent in all sample groups; ovaries in the “final maturation” and “post-spawning” categories appear to exhibit inversely proportional lines, while “hardened” ovaries were most frequent in group G3. For **b** and **c**, different letters indicate a significant difference between the groups

The tilapia reproductive cycle is classified as continuous with multiple-batch spawning (Barbieri et al. 2018), which is directly reflected in its ovarian morphology throughout the cycle. Macroscopic analysis demonstrated three distinct ovarian patterns based on coloration, consistency, presence of visible structures, and morphometric parameters (weight [wt], length [L], and width [W]), which we defined as “final maturation,” “post-spawning,” and “hardened” (Fig. 3a). The ovarian characteristics we observed are in line with the reproductive cycle, especially the presence of ovaries classified as “final maturation” and “post-spawning,” stages which are expected in species with multiple spawnings (Wallace and Selman 1981; Coward and Bromage 2000; Silva 2019).

In more detail, ovaries classified as “final maturation” were voluminous (wt̅ = 18.79 g, L̄ = 5.86 cm, W̄ = 1.87 cm), intense yellow in color and filled with large, spherical, individualized oocytes occupying practically all the internal space, indicating imminent readiness for spawning. The ovaries classified as “hardened” were firm and full (wt̅ = 23.20 g, L̄ = 5.87 cm, W̄ = 1.85 cm), and intense yellow with reddish/purplish areas. We found no descriptions or reports of this ovarian pattern in the literature on fish, which hampered physiological interpretation and reinforces the need for more in-depth investigations. The hardened consistency and color changes suggest degenerative processes, potentially related to advanced atresia, tissue degeneration, and/or oocyte necrosis, which may be associated with external factors such as management conditions in the fish farming system. Ovaries classified as “post-spawning” were smaller (wt̅ = 7.51 g, L̄ = 5.84 cm, W̄ = 1.86 cm), translucent red with a watery appearance and the presence of small and barely visible oocytes along with atretic oocytes and reabsorbing residues, typical characteristics of the post-spawning period in teleost fish (Grier et al. 2009; Brown-Peterson et al. 2011).

In the statistical morphometric analysis, only weight differed significantly in the three ovarian pattern classifications (*H* = 60.107, *p* < 0.001; Fig. 3b), with variation observed in comparisons between the “post-spawning” group and the other remaining groups (“final maturation:” *W* = 4.927, *p* < 0.001; “hardened:” *W* = 3.110, *p* = 0.002). This is expected in a species with multiple-batch spawning, since the accumulation and successive release of oocytes directly influences ovarian volume and weight throughout the reproductive cycle (Tyler and Sumpter 1996; Vazzoler 1996; Brown-Peterson et al. 2011).

In addition to ovary weight by class, we also analyzed the gonadosomatic index (GSI, gonad weight: individual’s total weight). Since GSI is widely used to infer reproductive investment (Sturm 1978; Abdalla et al. 2024), interpretating this indicator depends on the homogeneity of body weight among the analyzed groups and requires verification of total female weight distribution among the sampled groups. We found a significant difference between the weights of the females (*H* = 22.137, *p* = 0.002), but only in the control group (G0) compared to the others (W_G0-G1_ =  − 2.987, *p* = 0.003 / W_G0-G2_ =  − 2.334, *p* = 0.020 / W_G0-G3_ =  − 2.616, *p* = 0.009 / W_G0-G4_ =  − 3.342, *p* = 0.001 / W_G0-G5_ =  − 3.541, *p* < 0.001 / W_G0-G6_ =  − 2.987, *p* = 0.003 / W_G0-G7_ =  − 2.800, *p* = 0.005). For this reason, we excluded the GSI value for group G0 from the analysis with the other groups, since it does not actually reflect a valid proportional comparison with the other groups and its inclusion would lead to methodological biases.

When the control group was excluded, the GSI value varied significantly between the remaining groups (*H* = 18.362, *p* = 0.005; Fig. 3c), between group G2 and groups G3 to G7 (W_G2-G3_ = 2.616, *p* = 0.009 / W_G2-G4_ = 2.616, *p* = 0.009 / W_G2-G5_ = 3.179, *p* = 0.001 / W_G2-G6_ = 2.535, *p* = 0.011 / W_G2-G7_ = 2.656, *p* = 0.008) and also between group G1 and groups G5 and G7 (W_G1-G5_ = 2.352, *p* = 0.019 / W_G1-G7_ = 2.091, *p* = 0.037). The remaining groups did not exhibit significant differences (*p* > 0.05). This pattern is consistent with work on other teleosts (Trujillo-Jiménez et al. 2013; Veranes-Dip et al. 2022) which has shown that GSI increases progressively alongside gonad development and the transition to more advanced reproductive stages, indicating greater energy allocation to the gonads. The lack of significant differences between some intermediate groups suggests phases of relative stability in reproductive investment, a finding which has also been reported in comparative studies on species with continuous or seasonal reproductive cycles (Stéquert et al. 2001; Santos Schmidt et al. 2021).

Our GSI results may be related to the frequency distribution of the analyzed ovarian patterns; this was expected, since significant differences in weight were seen among the ovarian classification groups. As for the frequency of each ovarian pattern (Fig. 3d), ovaries classified as “final maturation” predominated in all the sample groups, ranging from 50 to 75%, while “post-spawning” and “hardened” ovaries accounted for 10–40% and 0–15% of the ovaries per group, respectively. More detailed analysis found inversely proportional relationships between the frequencies of ovaries classified as “final maturation” and “post-spawning,” especially in groups G1 and G6, where “final maturation” ovaries were significantly less frequent as “post-spawning” ovaries increased significantly. This inversely proportional relationship suggests clear progression in the reproductive cycle, indicating the recent occurrence of reproductive events in these groups and reflecting the transition between the pre-ovulatory phase and the period following oocyte release (Vazzoler 1996; Brown-Peterson et al. 2011; Cassel et al. 2017a).

Studies have described this phenomenon in teleost fish, with ovaries classified as “final maturation” decreasing after spawning while “post-spawning” ovaries became more evident, characterized by the presence of atretic follicles and POCs (Cassel et al. 2017a; Corriero et al. 2021; Shimizu et al. 2021). The occurrence of this pattern in distinct groups, such as G1 and G6, may indicate asynchrony throughout the sampling period, consistent with species like Nile tilapia that spawn in batches or have prolonged reproductive cycles (Pereira et al. 2013; Barbieri et al. 2018). The alternation in the frequencies of these ovarian patterns consequently reinforces the interpretation that the groups analyzed in this research represent different moments in the reproductive cycle, corroborating the use of the distribution of ovarian classes as a reliable indicator of reproductive activity.

“Hardened” ovaries were most frequent in group G3, representing approximately 15% of all ovaries collected, and were less common in the other groups, suggesting that this is not a common physiological condition within the species’ reproductive cycle. This pattern may be related to specific management factors or pathophysiological alterations that have not yet been described for Nile tilapia. Within this context, histological analyses and descriptions are indispensable for correct identification of oocyte stages and the elucidation of processes such as atresia, necrosis, or other alterations in germinal tissues.

### Microscopic characteristics of the ovaries and involution processes

**Fig. 4:**
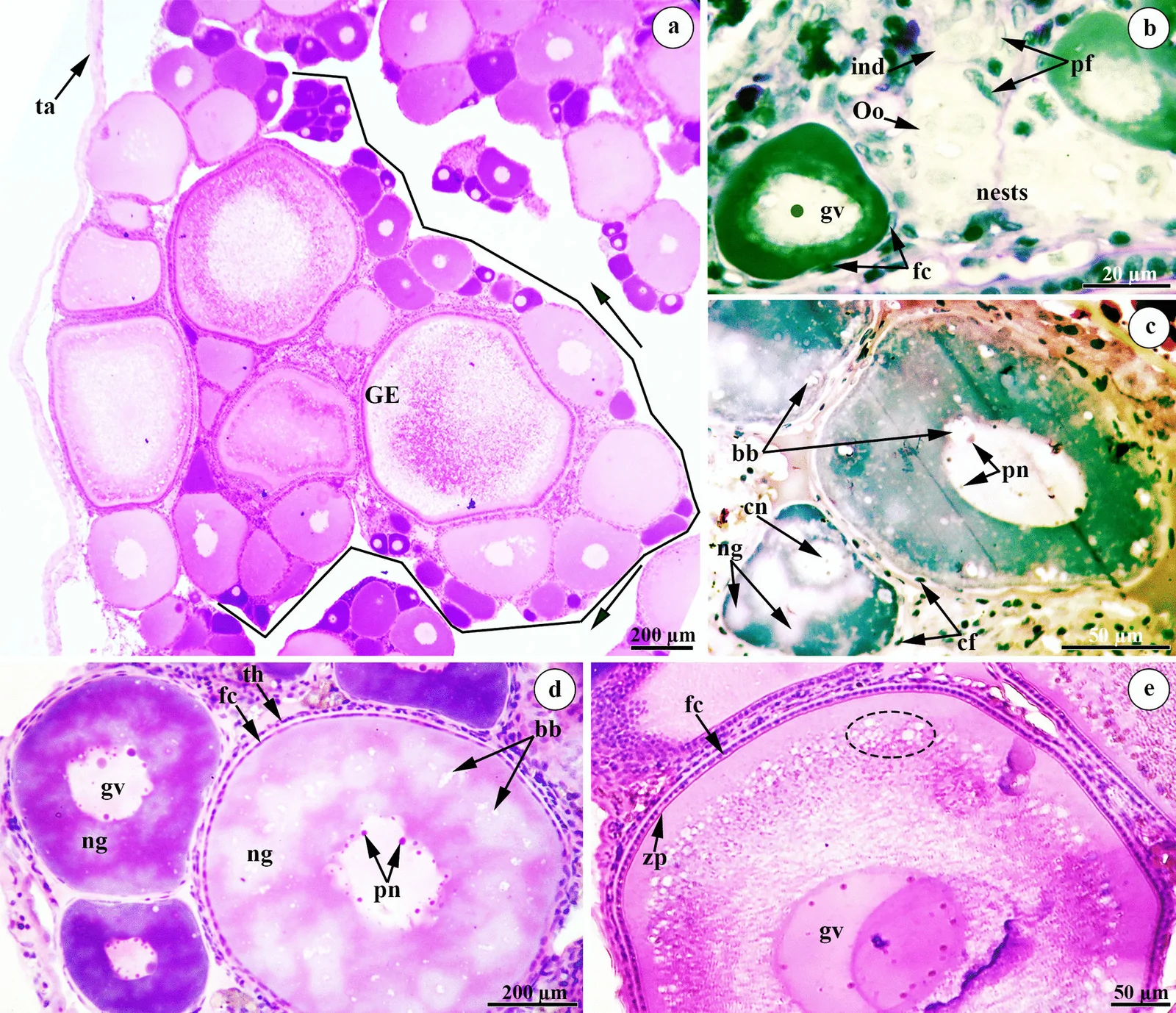
Histological composition of Nile tilapia ovaries. **a** The ovaries are of the cystovarian type, with a tunica albuginea (ta) that emits connective tissue septa into the interior of the organ (arrows), delimiting the ovarian lamellae (line) and the germinal epithelium (GE). **b** Oogonia (Oo) have clear cytoplasm and are generally grouped into nests surrounded by prefollicular cells (pf); next, they are individualized (ind) by the prefollicular cells. After differentiation into early oocytes, these follicles undergo several changes in the germinal vesicle (gv), the cytoplasm [with the identification of nuages (ng) and Balbiani bodies (bb)], and the surrounding layers [with the development of follicular cells (fc)], namely: **b** single central nucleolus follicles, **c** multiple central nucleoli (cn) follicles, and **c**, **d** perinucleolar (pn) follicles; in the latter there is also the development of the theca layer (th). The end of primary growth is marked by cortical alveoli stage follicles (**e**), with the appearance of cortical alveoli (dashed circle), cuboidal follicular cells (fc), and initial development of the zona pellucida (zp). Staining: **a**, **d**, **e** HE; **b**, **c** PAS + ferric hematoxylin + metanil yellow

The histological organization of Nile tilapia ovaries in the sampled individuals exhibited typical characteristics for cystovaries (Fig. 4a). A thick tunica albuginea surrounds the ovaries, with connective tissue septa that project into the organ to organize and delimit the ovarian lamellae. Along with the connective tissue septa, these lamellae are comprised of the germinal epithelium, in which germ cells in different stages of development can be observed.

Cystovarian-type ovaries are widely described in the literature for teleost fish with external fertilization and continuous, multiple-batch, and/or indeterminate spawning, where oocytes are released into the ovarian lumen and conducted through continuous oviducts toward the external environment where fertilization occurs, promoting greater availability of gametes throughout the reproductive period (Grier et al. 2009; Cassel et al. 2017a). This structural organization suggests a fundamental role in mechanical support and adequate nutrient supply to follicles at different stages of development, essential aspects for oocyte growth and maturation (Grier et al. 2009). These morphological characteristics reinforce the functionality of the cystovarian-type ovary in Nile tilapia, and are consistent with the continuous and multiple-batch spawning reproductive model described for this species (Barbieri et al. 2018).

Oogonia constitute the primary germ cell population and represent the initial stage of oocyte development, ultimately culminating in the ovulation process. In our study model, these cells had nearly translucent cytoplasm (Fig. 4b) and were generally located near larger oocytes or grouped into nests surrounded by prefollicular cells. These oogonia can divide by mitosis (forming cell nests) or can commit to the formation of mature follicles that will later be released; in this latter case, they become individualized (Fig. 4b) by the prefollicular cells and begin to receive stimuli for cell differentiation. The transition from oogonia to early oocytes, marked by cellular individualization in prefollicular cells and the emergence of evident nuclei with central nucleoli, represents the beginning of meiosis and oogenesis (Grier et al. 2009; Lubzens et al. 2010; Cassel et al. 2017a).

After differentiation into early oocytes, these cells develop well-defined spherical nuclei containing a single evident central nucleolus (Fig. 4b). From this point on, the nucleus is called the germinal vesicle. At this stage, the prefollicular cells begin to organize themselves as follicular cells, remaining connected to the germinal epithelium, and the follicle begins primary growth. During the progression of oocyte development, follicles with multiple central nucleoli were observed (Fig. 4c), and subsequently with peripherally distributed nucleoli (perinucleolar follicles; Fig. 4c, d), maintaining the predominantly spherical germinal vesicle. In the cytoplasm, structures with regions of lower staining intensity compatible with nuage bodies were identified, along with Balbiani bodies, which appeared as vacuolated areas (Fig. 4c, d). Both structures became more evident in perinucleolar oocytes, accompanied by cytoplasmic expansion and the irregular outline of the germinal vesicle (Fig. 4d). At this stage, formation of the theca layer outside the follicular cells could also be observed (Fig. 4 d).

As the cytoplasm expanded, the emergence of cortical alveoli was identified (Fig. 4e), while the follicular cells assumed a cuboidal morphology and more defined organization characterizing the cortical alveoli stage follicles. We also observed the initial development of the zona pellucida, a complex structure characterized by the presence of transverse striations (similar to small rays or channels) compatible with microvilli that interconnect the oocyte to the follicular cells. At the end of primary growth, the germinal vesicle again presented a regular spherical outline.

**Fig. 5:**
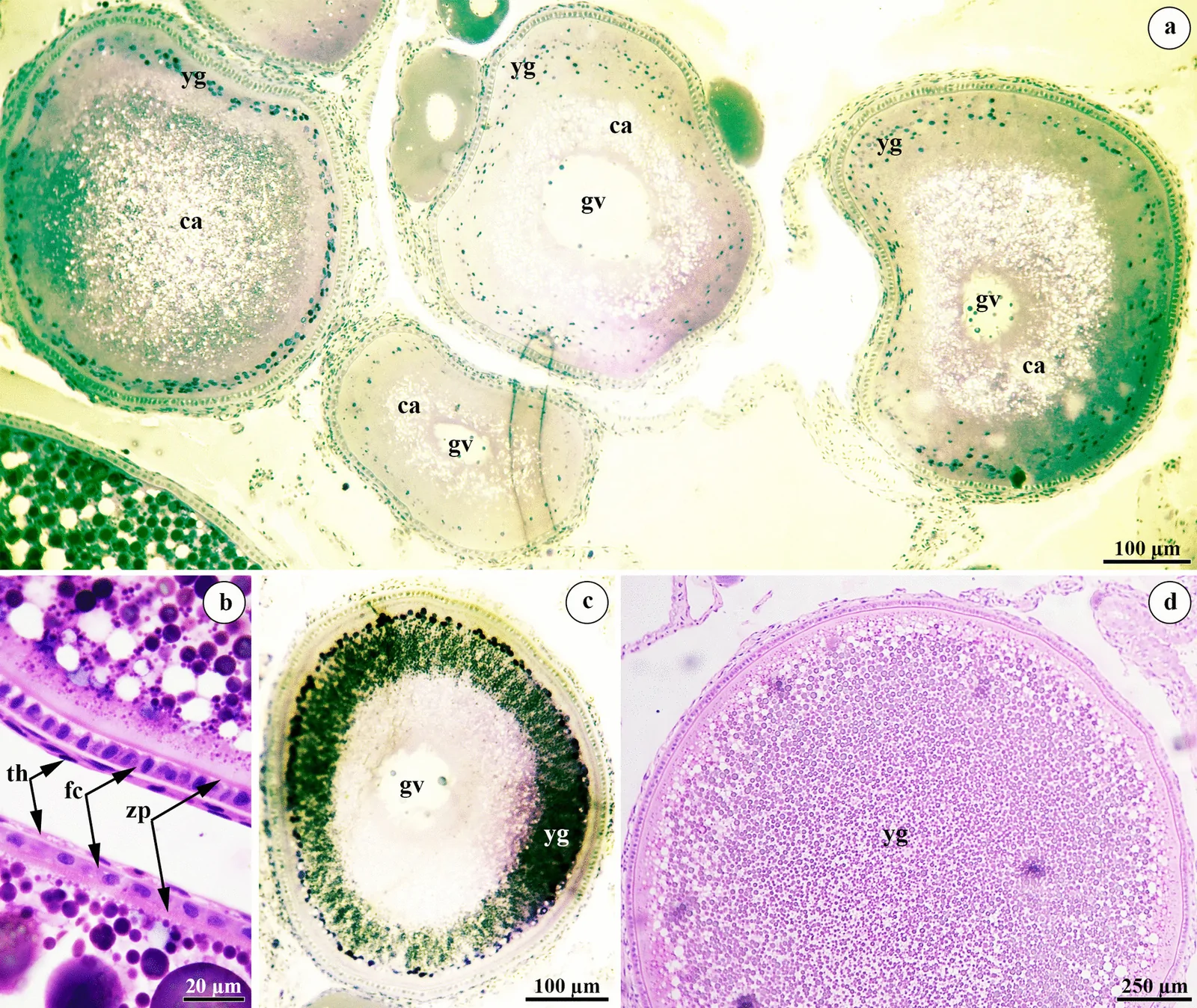
Secondary growth of ovarian follicles in Nile tilapia. **a** The follicles show a progressively more well-defined germinal vesicle (gv); there is also greater evidence of the cortical alveoli (ca) and the start of cytoplasmic filling by yolk granules (yg). **b** The development of the theca cell layer (th) is evident, the follicular cells (fc) maintain a cuboidal morphology, and there is greater development of the zona pellucida (zp). Gradual accumulation of yolk (yg) in the oocyte leads to the identification of early (**a**), intermediate (**c**), and mature (**d**) secondary follicles. Staining: **a**, **b** PAS + ferric hematoxylin + metanil yellow, **c**, **d** HE

In secondary growth (Fig. 5), the follicles began to present a well-defined, spherical germinal vesicle with peripherally distributed nucleoli. At this stage, greater evidence and development of the cortical alveoli (Fig. 5a), zona pellucida, and theca cell layer (Fig. 5b) were observed, while the follicular cells maintained cuboidal morphology (Fig. 5b) and cytoplasmic filling with yolk began to be visible. This gradual accumulation of yolk in the oocyte marks the secondary growth stage, and we identified intermediate secondary follicles, in which part of the cytoplasm’s volume was occupied by yolk granules (Fig. 5c), and mature secondary follicles (Fig. 5d), characterized by cytoplasm largely filled with yolk.

The presence of oocytes in different stages of follicular growth, from the multiple nucleolus phase to complete maturation, reflects the continuous dynamics of folliculogenesis in Nile tilapia, teleost fish with external fertilization and indeterminate fecundity (Selman and Wallace 1989; Lubzens et al. 2010). During this process, oocyte development occurs in a coordinated manner with the action of follicular and theca cells, which provide essential structural, metabolic, and endocrine support for oocyte growth and differentiation (Lubzens et al. 2010).

Notable among the structures described is a zona pellucida which is well-defined even in the primary growth stage. The early formation of this structure, associated with the presence of cortical alveoli, suggests a possible early activation of mechanisms related to the synthesis, transport, and deposition of proteins and glycoproteins, marking the transition to more advanced stages of oocyte development. These events depend directly on communication between the oocyte and follicular cells mediated by specialized junctions, which are fundamental for efficient vitellogenesis and oocyte maturation (Grier 2000; Grier et al. 2009; Lubzens et al. 2010; Cassel et al. 2017a).

During secondary growth, progressive accumulation of yolk granules in the cytoplasm characterizes the vitellogenic phase, storing and supplying energy reserves that will sustain embryonic development after fertilization (Grier et al. 2009; Quagio-Grassiotto et al. 2011; Cassel et al. 2017a). This accumulation progresses until complete cytoplasmic filling in the maturation phase (Honji et al. 2006; Lubzens et al. 2010; Quagio-Grassiotto et al. 2011; Cassel et al. 2017a), reinforcing the functionality and efficiency of the reproductive process in this species.

Once it is ready, the mature follicle can then be ovulated. This involves the rupture of surrounding layers, through which the oocyte is displaced and moved into the ovarian lumen; the cells remaining after this release form the POC (Grier et al. 2009; Lubzens et al. 2010). Meanwhile, oocytes that were not released during the reproductive phase undergo the process of degeneration and reabsorption known as follicular atresia (Santos et al. 2008; Cassel et al. 2017b). Details and images of these involution processes (follicular atresia and POCs) are presented in the following sections.

Taken together, our histological findings for *O. niloticus* are consistent with the model of gradual, asynchronous, and finely regulated ovarian development described for tropical teleosts. The stages of oocyte development described above, along with their main histological characteristics, are summarized in Table 1. The coexistence of different stages of follicular development reinforces the reproductive plasticity of this species and supports its efficient reproductive strategy, contributing to the high reproductive success observed in natural and cultivated environments.

**Table 1:** Main histological characteristics of the oocyte development stages of Nile tilapia

Oocyte stages	Main histological characteristics
Oogonia	First germ cells, with clear/translucent cytoplasm; organized in nests and surrounded by prefollicular cells
Early oocyte	An oocyte is individualized by prefollicular cells, with a spherical nucleus (germinal vesicle) and a single central nucleolus; prefollicular cells are then called follicular cells
Primary growth	Multiple central nucleoli follicles and perinucleolar follicles: present a spherical germinal vesicle, nuages (less stained regions), and Balbiani bodies (vacuolated areas); these characteristics are more evident in perinuclear follicles, along with development of the theca cell layer Cortical alveoli stage follicles: appearance of cortical alveoli, cuboidal follicular cells, and zona pellucida with transverse striations
Secondary growth	Follicles show a well-defined germinal vesicle, greater evidence of the cortical alveoli and the start of cytoplasmic filling by yolk granules; gradual accumulation of yolk in the oocyte leads to the identification of early, intermediate, and mature secondary follicles

### Characteristics of the involution process of follicular atresia

Follicular atresia is an essential physiological process for maintaining metabolic balance and regulating fertility in females (Wallace and Selman 1981; Santos et al. 2005, 2008; Grier et al. 2009). This process also contributes to energy recovery and adaptation to variations in physiological and environmental conditions (Wallace and Selman 1981; Guraya 1986; Santos et al. 2005, 2008; Grier et al. 2009; Cassel et al. 2017b). In multiple-batch spawning species like Nile tilapia this mechanism is especially important, since it permits dynamic adjustment of the reproductive cycle through reabsorption of the nutrients in oocytes that do not fully complete the maturation process (Cassel et al. 2017b; Babu et al. 2025).

**Fig. 6:**
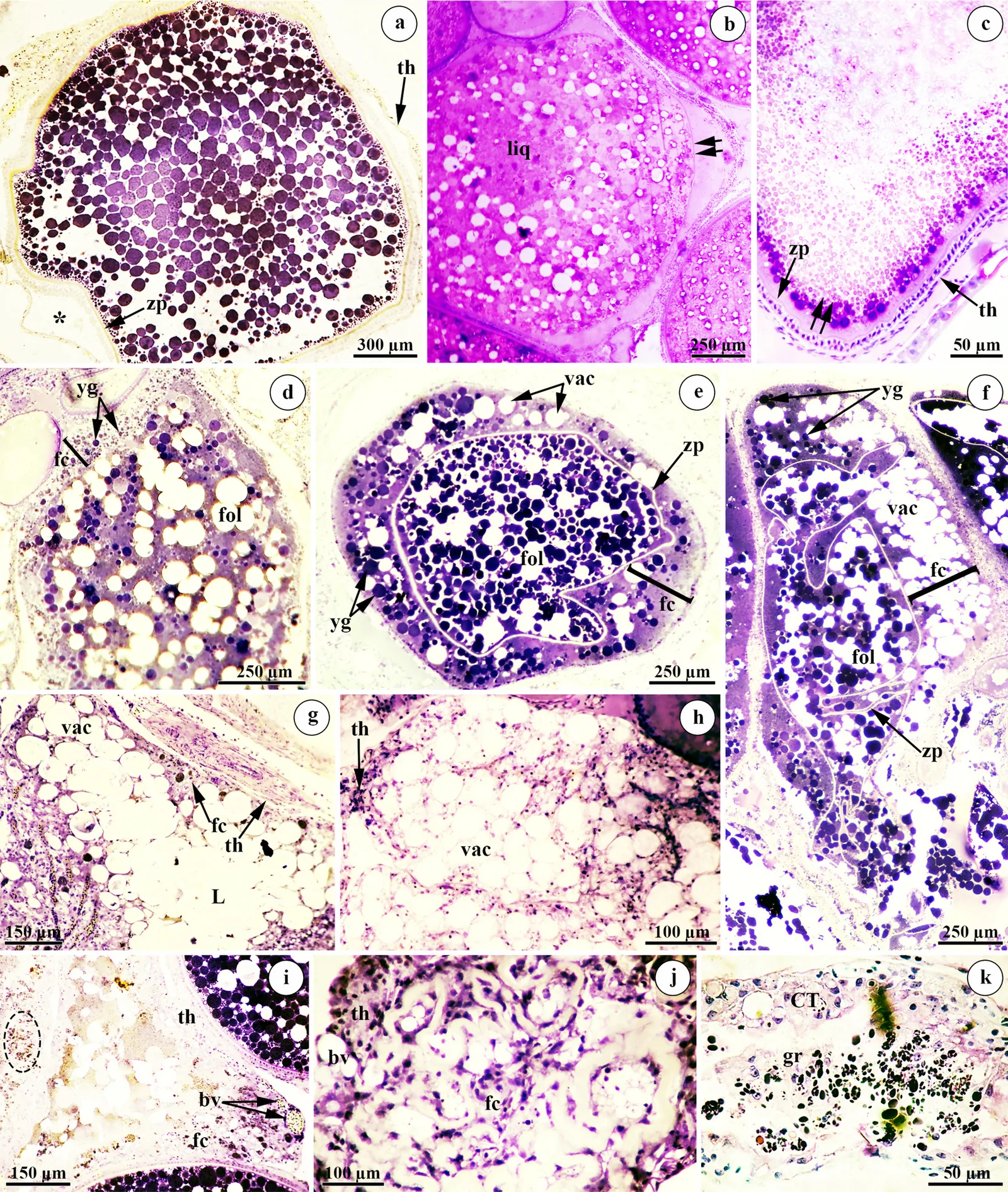
Involution process of follicular atresia in Nile tilapia ovaries. **a**–**c** The process begins with retraction (double arrows) and liquefaction (liq) of the yolk granules, the zona pellucida (zp) appears progressively irregular, and the theca cell layer (th) detaches (*). **d**–**f** Next, a progressive reduction in follicular volume (fol) is observed, along with increased irregularity of the zona pellucida (zp) and cylindrical follicular cells (fc) with yolk granules (yg) in their cytoplasm; with the digestion of these granules, cytoplasmic vacuoles (vac) begin to appear. **g**, **h** As it progresses, the lumen (L) reduces or closes, and a vacuolated cell aggregate (vac) delimited by the theca layer (th) is formed. **i**–**k** Finally, there is a reduction in vacuoles in the follicular cells (fc), which become mixed with the theca cells (th), with more evident blood vessels (bv), as well as the presence of brownish granules (dashed circle, gr) in the tissue; these structures intermingle with the adjacent connective tissue (CT) until complete reabsorption. Staining: **a**, **d**–**k** PAS + ferric hematoxylin + metanil yellow, **b**, **c** HE

The involution process of follicular atresia in Nile tilapia is initially characterized by retraction of yolk granules in the cortical region of the oocyte, followed by yolk liquefaction (Fig. 6a–c). Simultaneous changes were also observed in the zona pellucida, follicular cells, and the theca cell layer. The zona pellucida exhibits a progressively irregular outline, indicating areas of yolk extravasation towards the follicular cells, which initially remained intact with cuboidal morphology. The follicular cells next begin to exhibit phagocytic characteristics, shifting toward cylindrical morphology and with cytoplasmic content containing yolk granules. As the yolk granules are incorporated and digested, the follicular cells begin to present intracytoplasmic vacuoles. The theca cell layer also appears detached from the follicular cells early in the atresia process. This separation does not seem to intensify until the end of the atresia involution process, but this layer appears to become thicker, possibly associated with increased vascularization.

The initial changes characterized by retraction and subsequent liquefaction of the yolk indicate the beginning of the oocyte reabsorption process, and are considered important morphological markers for the initial identification of follicular atresia in teleosts (Cassel et al. 2017b; Medina et al. 2021; Chikhoune and Ichalal 2024). According to Cassel et al. (2017b) and Medina et al. (2021), yolk degradation occurs before the structural collapse of the follicle, demonstrating that the oocyte is the first structure to undergo degenerative changes during the atresia process.

The zona pellucida also exhibits morphological changes, characterized by a loss of contour regularity and the appearance of fissures. This process favors the phagocytosis of yolk material by follicular cells, and is described in the literature on teleosts as a key point in the follicular reabsorption mechanism (Santos et al. 2005, 2008; Cassel et al. 2017b; Medina et al. 2021; Babu et al. 2025). Follicular cells also play an active role during the atresia process, serving as important metabolic elements (Cassel et al. 2017b; Medina et al. 2021; Chikhoune and Ichalal 2024). The observed morphological modifications, especially those associated with yolk granules and the presence of cytoplasmic vacuoles, indicate intense activation of lysosomal activity, a characteristic frequently described in fish (Miranda et al. 1999). These findings reinforce the relevance of follicular cells in the follicular involution process, demonstrating that their function goes beyond simple structural support as they assume a central role in the reabsorption and reuse of oocyte components.

Our results showed the progression of atresia (Fig. 6d–f) through continuous yolk reabsorption, accompanied by increased irregularity of the zona pellucida and progressive reduction of follicular volume. When all the yolk is reabsorbed and digested (Fig. 6g, h), only the presence of vacuoles in the follicular cells is observed, along with significant reduction of the lumen where the oocyte should be. As the atretic process progresses the lumen closes, forming a vacuolated cell aggregate which is still delimited by the theca cells. During the final stages (Fig. 6i–k), a reduction in cytoplasmic vacuoles is visible in the follicular cells, which begin to exhibit degenerative characteristics (process of death and reabsorption) and become indistinguishable from the theca cells. At this stage, blood vessels become more evident, along with the presence of brownish granules in the tissue, consistent with the accumulation of residual pigments, probably related to lipofuscin granules present in cell death processes (Cassel et al. 2017b; Babu et al. 2025). At the end of the process, these structures show a progressive reduction in size, intermingling with the adjacent connective tissue until complete reabsorption.

The final stages of the atretic process are characterized by the progressive reduction of the follicular lumen and formation of vacuolated cell aggregates, which reflect a marked disorganization of the remaining follicular structural tissue (Cassel et al. 2017b; Medina et al. 2021; Babu et al. 2025). At this stage, it becomes impossible to distinguish between follicular cells and theca cells, indicating an advanced degree of tissue resorption, where the presence of brown-colored granules (possibly corresponding to lipofuscin) reinforces the occurrence of cell death, an event widely associated with tissue degeneration processes in vertebrates (Guraya 1986; Santos et al. 2005, 2008; Cassel et al. 2017b; Medina et al. 2021).

The follicular atresia stages observed in this study were classified according to the pattern described for teleosts, where atresia corresponds to a progressive and ordered process of oocyte degeneration (Miranda et al. 1999; Melo et al. 2014; Cassel et al. 2017b; Medina et al. 2021; Chikhoune and Ichalal 2024; Babu et al. 2025), but morphological characteristics specific to Nile tilapia were also considered. We classified follicular atresia into four stages: initial, intermediate, advanced, and final (Table 2). Initial atresia is characterized by liquefaction of the yolk granules, fragmentation of the zona pellucida, and detachment of the theca cell layer. In the intermediate stage, we can observe the presence of follicular cells with phagocytic activity which were involved in the yolk reabsorption process. Advanced atresia is characterized by reduction or closure of the lumen, with the formation of a vacuolated cell aggregate still delimited by the theca cell layers. The final stage is marked by a reduction in the number of follicular and theca cells, as well as the presence of lipofuscin granules and granulocytes near the atretic follicle.

**Table 2:** Main histological characteristics of the involution process of follicular atresia in Nile tilapia ovaries

Atresia stages	Main histological characteristics
Initial	Shrinkage and liquefaction of yolk granules, irregular outline of the zona pellucida, and detachment of the theca cell layer
Intermediate	Columnar follicular cells with phagocytic activity, contributing to the reabsorption of yolk granules
Advanced	Reduction or closure of the lumen, formation of a vacuolated cell aggregate delimited by the theca cell layer
Final	Reduction in follicular and theca cells, presence of lipofuscin granules and nearby granulocytes

The structural changes we observed mostly follow patterns which are described in the literature for atretic processes (Grier et al. 2009; Melo et al. 2014; Cassel et al. 2017b; Medina et al. 2021; Chikhoune and Ichalal 2024). This present study, however, makes a significant contribution to the ovarian histology of Nile tilapia by expanding the morphological classification of follicular atresia, especially by describing alterations in the theca layer and evidence of cell death visible under light microscopy. These findings reinforce the importance of follicular atresia as an essential mechanism for the reproductive adaptation and success of this species.

### Characteristics of the involution process of post-ovulatory complexes (POCs)

**Fig. 7:**
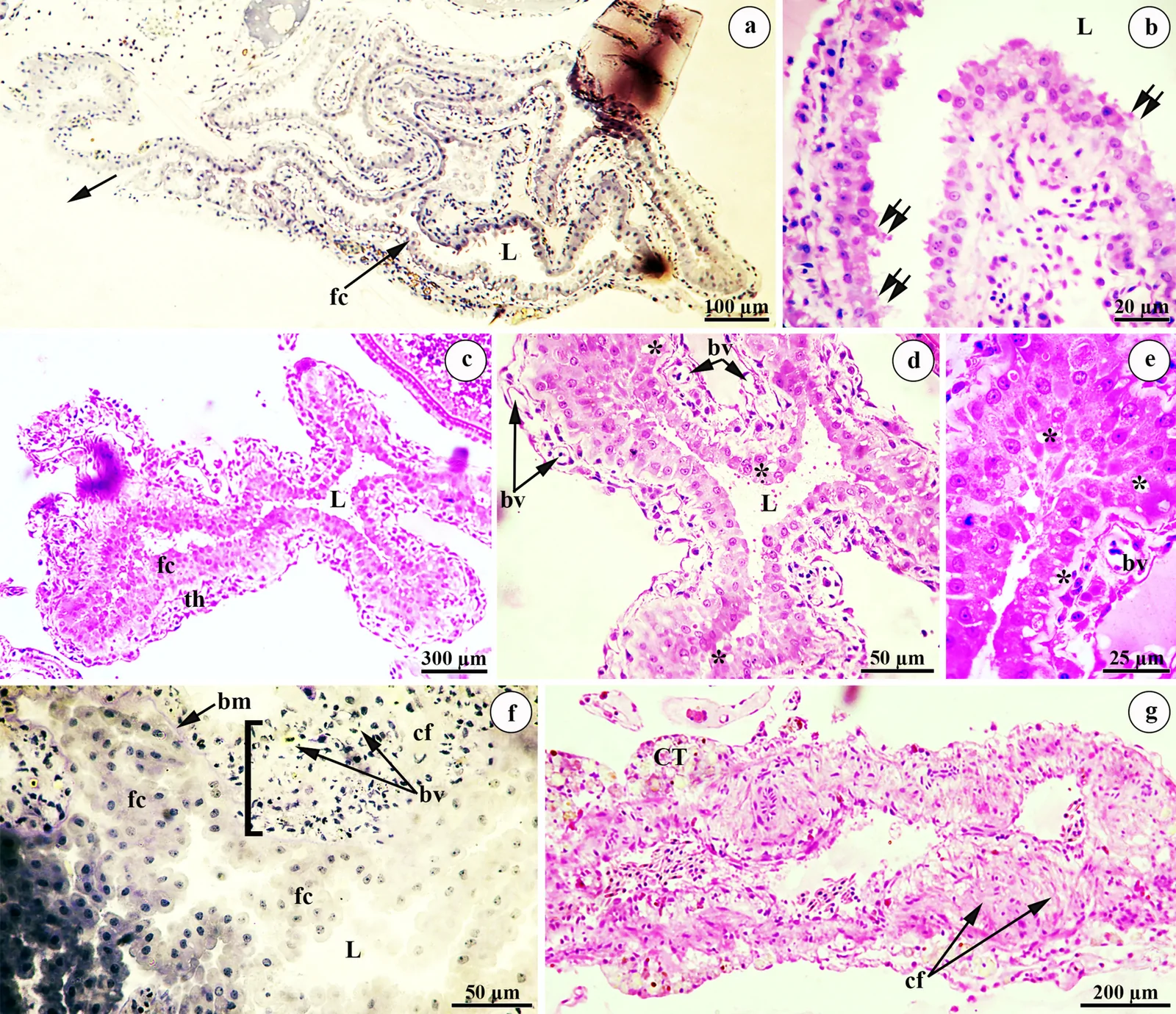
Involution process of post-ovulatory complexes (POCs) in Nile tilapia ovaries. **a**, **b** For ovulation to occur, the surrounding layers are ruptured and create an opening (arrow); at this time, the follicular cells (fc) are cuboidal and have cytoplasmic projections (double arrows) directed towards the lumen (L). **c**–**e** Subsequently, gradual reduction of the lumen (L), decrease/absence of cytoplasmic projections of the follicular cells (fc), and the appearance of vacuoles (*) in their cytoplasm can be seen; the blood vessels (bv) of the theca layer (th) become more evident. **f** As it progresses, the lumen (L) is almost completely closed, and the follicular cells (fc) detach from the basement membrane (bm); the basement membrane and the theca cell layer (bracket) appear thickened, as connective tissue fibers (cf) and blood vessels (bv) are more present. **g** Finally, a cell cluster remains immersed in the adjacent connective tissue (CT) until complete reabsorption. Staining: **a**, **f** PAS + ferric hematoxylin + metanil yellow; **b**–**e**, **g** HE

Surrounding layers are ruptured for ovulation to occur, creating an opening through the layers of follicular cells, basement membrane, and theca cells; the layers that are not released comprise the POCs (Fig. 7a), which also result from the reorganization and involution of the follicular structures that remain after the release of an oocyte (Melo et al. 2014; Cassel et al. 2017b; Ferreri et al. 2021; Chikhoune and Ichalal 2024). This process has been widely described in the literature on teleost fish, since it represents a fundamental phase of the ovarian cycle, allowing the reabsorption and removal of tissues that do not participate in the ovulatory process and contributing to proper adjustment of the reproductive cycle (Drummond et al. 2000; Kumar and Joy 2015; Ferreri et al. 2021).

During the initial stages (Fig. 7a, b), the follicular cells feature cuboidal morphology, with cytoplasmic projections directed towards the lumen, while the basement membrane and the theca cell layer do not exhibit evident alterations. According to Wallace and Selman (1981), the characteristics observed in the initial phase suggest a recent post-ovulation stage, in which the follicular cells have not yet initiated degenerative processes. The absence of evident modifications in the basement membrane and theca reinforces the hypothesis that the initial reorganization occurs predominantly within the follicle; additionally, the cuboidal morphology of the follicular cells is maintained, along with the cytoplasmic projections directed towards the lumen, indicating that these cells preserve their structural and functional organization at this initial stage.

As the involution process progresses (Fig. 7c–e), a gradual reduction in the lumen can be observed, accompanied by a decrease in the cytoplasmic projections of the follicular cells and the appearance of vacuoles in the cytoplasm. The blood vessels in the theca layer also become more evident, presenting a dilated appearance. These characteristics demonstrate the advancement of cellular degeneration processes and autophagic activity, since this pattern is related to intracellular digestion and metabolic reuse of tissue components (Guraya 1986; Cassel et al. 2017b; Babu et al. 2025). The reduction in cytoplasmic projections may also indicate a loss of cellular polarity and progressive structural disorganization (Guraya 1986; Cassel et al. 2017b). According to Drummond et al. (2000), the marked increase in vascularization may be associated with both transport of waste products resulting from tissue degradation and maintenance of residual endocrine activity characteristic of the post-ovulatory period.

As the involution process continues (Fig. 7f), the lumen appears almost completely closed, with the follicular cells appearing to disconnect from the basement membrane and beginning to organize themselves into a cell aggregate. The basement membrane and theca cell layer are also seen to thicken, a phenomenon associated with an increased concentration of connective fibers and their vascularization. In the final phase (Fig. 7f), the lumen is completely occluded, leaving a cell cluster integrated with the adjacent connective tissue and rich in connective fibers, which exhibited progressive reduction until it was entirely reabsorbed.

The progressive closure of the lumen, associated with the rearrangement of follicular cells into vacuolated aggregates, characterizes the final phase of POC reorganization (Drummond et al. 2000; Cassel et al. 2017b). The thickening of the basement membrane and theca layers, along with increased vascularization, consequently seems to indicate an active tissue response aimed at removal and reabsorption of remaining structures. Studies demonstrate that these patterns are consistent with previous descriptions in teleosts, where POCs undergo rapid involution and are completely reabsorbed by the adjacent connective tissue (Miranda et al. 1999; Drummond et al. 2000; Grier 2000; Murua et al. 2006; Cassel et al. 2017b).

Within the literature, POCs are widely recognized as reliable histological markers of recent spawning in fish (Drummond et al. 2000; Grier 2000; Murua et al. 2006; Kumar and Joy 2015; Chikhoune and Ichalal 2024). However, most studies only identify the presence or absence of these structures, without delving into analysis of their temporal dynamics of involution. In this study, POCs were also classified into four stages of involution (Table 3), based on the microscopic characteristics described above. The initial stage presents cuboidal follicular cells, with cytoplasmic projections directed towards the lumen. In the intermediate stage, there is reduction of the lumen, the appearance of vacuoles in the cytoplasm of the follicular cells, and greater evidence of vascularization in the theca layers. The advanced stage is characterized by an almost completely closed lumen, follicular cells organized in vacuolated aggregates, as well as thickening of the basement membrane and theca layers. In the final stage, the lumen is completely occluded, leaving only a cluster of cells that will later be reabsorbed. The classification for POC involution stages proposed in this study is supported by previously established morphological descriptions for tropical species (Drummond et al. 2000; Grier 2000, 2012; Romagosa et al. 2005; Santos et al. 2005, 2008; Murua et al. 2006; Thomé et al. 2006; Grier et al. 2009; Melo et al. 2014; Cassel et al. 2017b), but represents an advance with a more detailed and rigorous systematization of degenerative processes, contributing to more precise interpretation of post-ovulatory dynamics.

**Table 3:** Main histological characteristics of the involution process of post-ovulatory complexes (POCs) in Nile tilapia ovaries

POCs stages	Main histological characteristics
Initial	Cuboidal follicular cells with cytoplasmic projections directed towards the lumen
Intermediate	Reduction of the lumen, appearance of vacuoles in the cytoplasm of follicular cells, and increased vascularization in the theca cell layer
Advanced	Almost completely closed lumen, follicular cells organized in vacuolated aggregates, thickening of the basement membrane and the theca cell layer.
Final	Lumen completely occluded, leaving a cluster of cells for later reabsorption

### Quantification of involutional processes: temporal analysis of the ovarian regressing process

The ovarian regressing phase is essential in the reproductive cycle of teleost fish, characterized by the reabsorption of oocyte structures as well as the remaining post-spawning structures from the previous cycle, and by the ovarian tissue remodeling necessary to begin a new reproductive cycle (Brown-Peterson et al. 2011; Grier 2012; Cassel et al. 2017a, b; Babu et al. 2025). In order to verify progression of the ovarian regressing process and indicate a temporal scale across the sampled groups, we quantified the involution stages of follicular atresia and POCs and compared these findings with data from mature secondary follicles. Since an abundance of mature follicles indicates that ovaries are ready for spawning, their decrease and replacement by involutional processes indicates post-spawning periods (Brown-Peterson et al. 2011; Cassel et al. 2017a). As for the ovarian regressing process, which is necessary to prepare for a new reproductive cycle by reabsorbing structures that will no longer be used and remodeling ovarian tissues (Brown-Peterson et al. 2011; Cassel et al. 2017a, b; Babu et al. 2025), an alternation between involutional structures and mature follicles is expected as the process progresses.

**Fig. 8:**
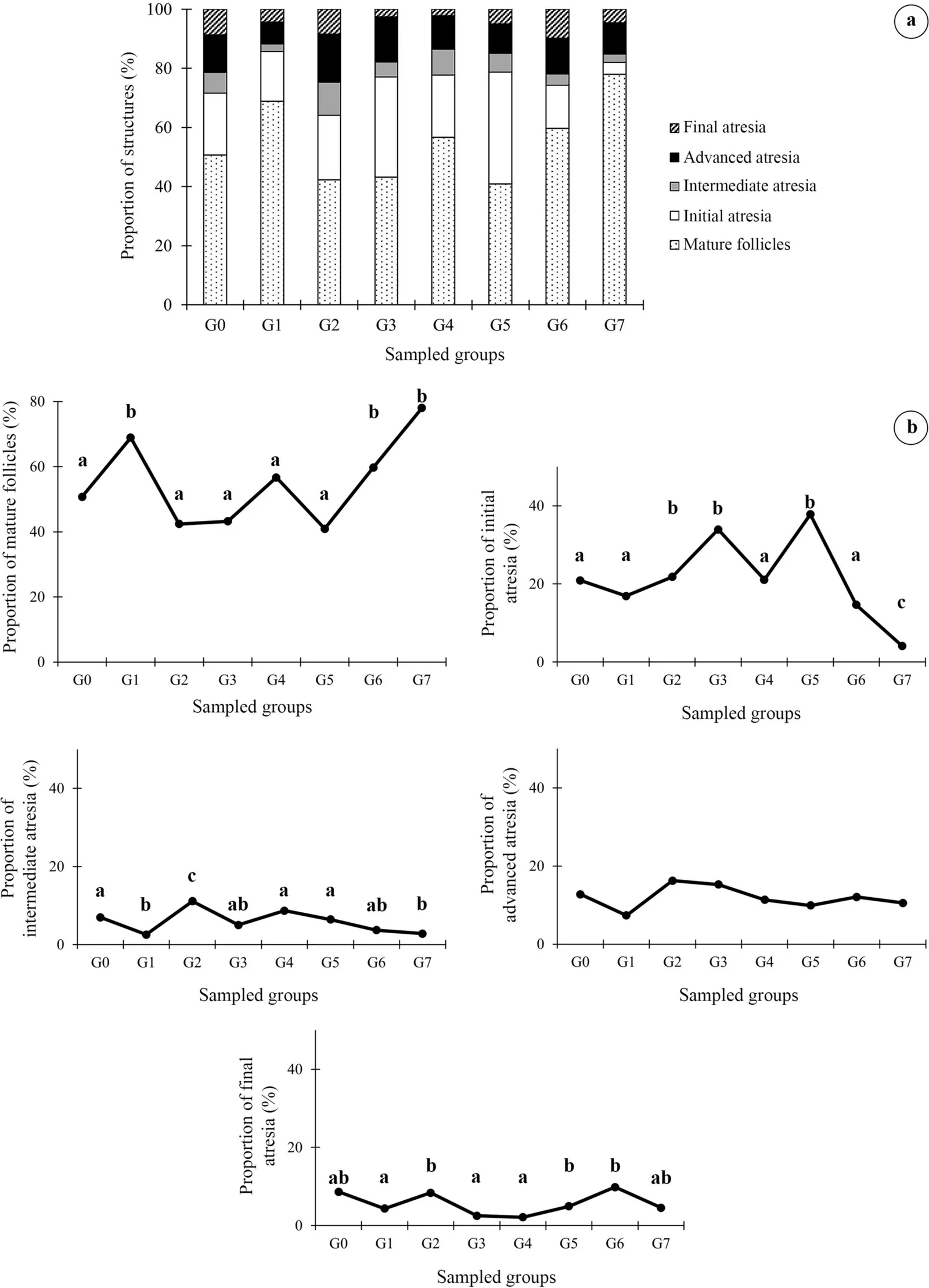
Quantitative analysis of the ovarian regression process, comparing the proportion of follicular atresia involution stages to the proportion of mature follicles across the sampled groups. **a** Distribution of the proportion of each structure in a single bar per group, totaling 100% of structures present in the ovaries. The same proportion is shown in **b**, but the variation in these values ​​for each of the structures in each group is evident; different letters indicate a statistical difference between the groups

Comparison of the quantitative data for the atresia stages with the findings for mature follicles (Fig. 8; Supplementary Table S1) showed a reduction in the proportion of mature follicles in groups G0 to G5, indicating a post-spawning period corresponding to the regressing phase. In groups G6 and G7 the proportion of mature follicles progressively increased, indicating a return of the ovaries for a new spawning cycle. Even after a reproductive cycle, the proportion of mature follicles did not fall below 40% (groups G0, G2 to G5), indicating their frequent presence even in spawned ovaries. The high frequency of mature follicles even during the ovarian regressing phase reinforces the asynchronous nature of ovarian development in Nile tilapia. Furthermore, group G1 showed a peak of mature follicles, even after de-mating: this event may be related to the persistence of vitellogenic oocytes that were not immediately ovulated, reflecting the asynchrony of the ovarian cycle (as highlighted earlier) and the overlap between the regressing and maturation phases. This pattern is widely described in species with multiple-batch spawning, where different oocyte stages develop simultaneously and allow for multiple reproductive events throughout the reproductive cycle (Lowerre-Barbieri et al. 2011; Barbieri et al. 2018). However, the reduction in the proportion of mature oocytes should not be interpreted as an isolated and definitive indicator of ovarian regression, since joint analysis of the involutional structures and other histological components of the ovary is necessary.

Considering the stages of follicular atresia, initial atresia was seen to be more prevalent in groups G2 to G5, with a slight decrease in group G4. Intermediate atresia was more frequent in group G2 and less prevalent in groups G3 to G6. The proportion of advanced atresia did not vary significantly among the sampled groups, while final atresia was more prevalent in groups G2, G5, G6, and G7. All stages of follicular atresia involution were less frequent in group G7. Progression of these atresia stages can be observed throughout the ovarian regressing process: considering G0 as the starting point (since the proportion of mature follicles was already low), the proportion of initial and intermediate atresia increased (in G2) and remained high until group G5 (despite some fluctuations). Final atresia was more frequent in G5 and G6, followed by a general decrease in the proportion of atresia and a significant increase in mature follicles in G7, when the ovaries appear to have recovered and were ready for a new cycle.

In teleost species like Nile tilapia with multiple-batch and continuous spawning, the regressing process occurs recurrently and is directly associated with the asynchronous development of oocytes, resulting in the coexistence of mature follicles within the same ovary along with structures in different stages of involution (Lowerre-Barbieri et al. 2011; Barbieri et al. 2018). Furthermore, the high frequency of atresia in the initial and intermediate stages during the post-spawning period reinforces the hypothesis that atresia acts as an important regulator of reproductive potential, allowing dynamic adjustments in response to physiological and environmental conditions (Murua et al. 2006; Cassel et al. 2017b; Charitonidou et al. 2020). Studies on Nile tilapia and other species that are important for aquaculture demonstrate that atresia does not necessarily constitute a pathological event but is rather a physiological component of the reproductive cycle, especially in species with continuous or prolonged cycles (Cassel et al. 2017b; Barbieri et al. 2018).

**Fig. 9:**
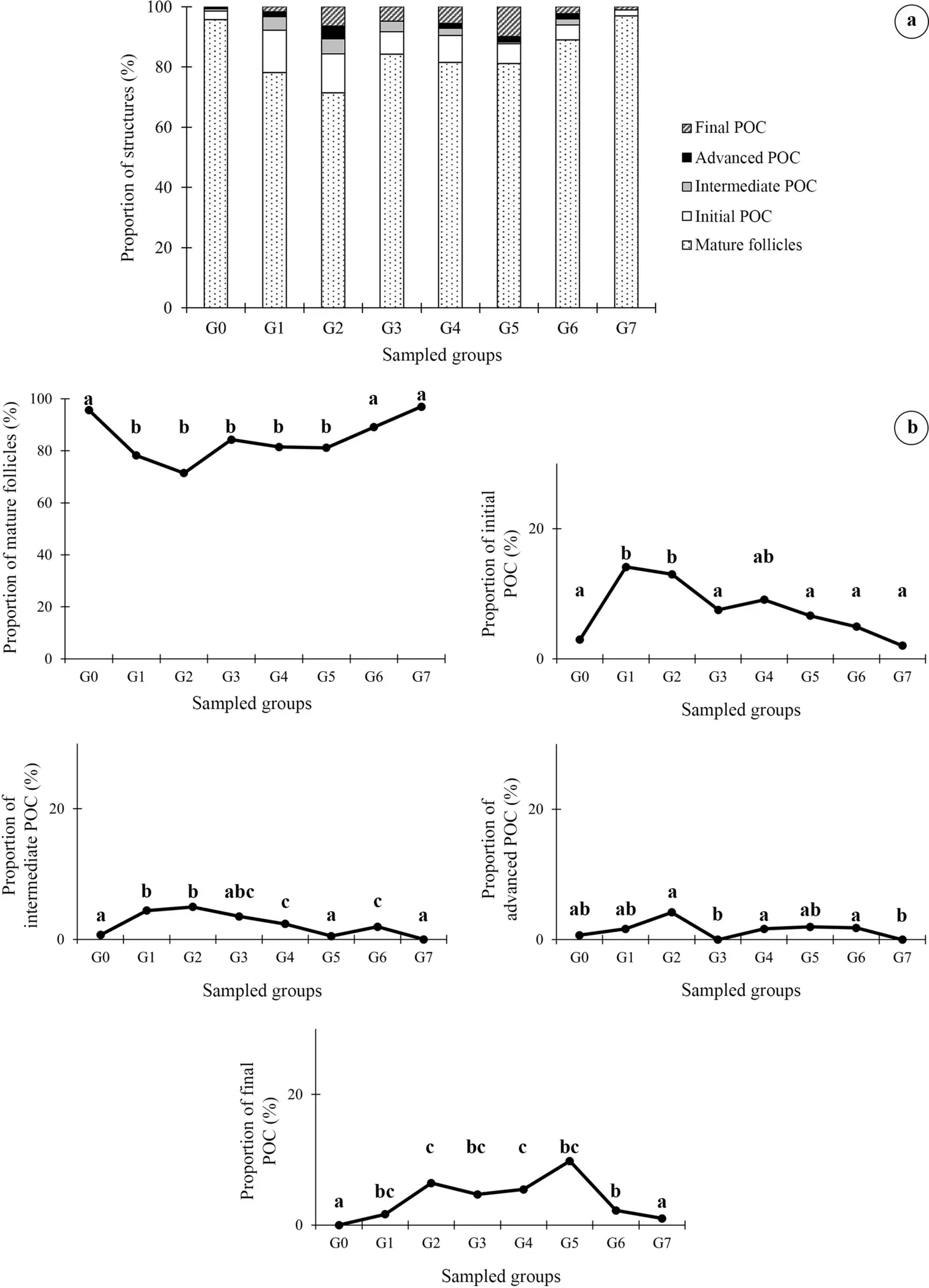
Quantitative analysis of the ovarian regression process, considering the proportion of post-ovulatory complex (POC) involution stages compared to the proportion of mature follicles across the sampled groups. **a** Distribution of the proportion of each structure in a single bar per group, totaling 100% of structures present in the ovaries. The same proportion is shown in **b**, but the variation in these values for each of the structures in each group is evident; different letters indicate a statistical difference between the groups

In a process similar to the quantitative analysis of atresia stages described above, we also compared the involution stages of POCs with the proportion of mature follicles across the sampled groups. A reduction in the proportion of mature follicles was observed in groups G1 to G5, indicating a post-spawning period, and in G6 and G7 the proportion of mature follicles increased progressively (Fig. 9; Supplementary Table S2). The post-spawning period is evidenced by verification of the POC involution stages (Fig. 9; Supplementary Table S2). Initial and intermediate POCs were more prevalent in groups G1 to G4, while initial POCs showed a slight decrease in group G3. Advanced POCs were not significantly abundant in any of the groups, although they clearly decreased in groups G3 and G7. Finally, the proportion of final POCs was higher in groups G2 to G5, and decreased progressively in groups G6 and G7.

In this way, progression of the involution stages of POCs throughout the ovarian regressing process could be seen. If we consider the peak of mature follicles in group G0 as the beginning of the regression process, a subsequent increase in the proportion of POCs was observed from the initial to final stages, maintaining high proportions until groups G4 and G5 (with some fluctuations). The presence of POCs decreases progressively in groups G6 and G7, coinciding with the progressive increase in the proportion of mature follicles and indicating that the ovaries are recovering for a new reproductive cycle.

In species with continuous reproduction, studies have demonstrated that POCs undergo a rapid reorganization process after spawning, and different stages of involution may coexist in the same ovary, reflecting the occurrence of multiple ovulatory events throughout a single reproductive cycle (Santos et al. 2005, 2008; Murua et al. 2006; Charitonidou et al. 2020). Furthermore, the progressive reduction in the frequency of POCs, concomitant with the increased proportion of mature follicles, indicates completion of the ovarian regressing process and functional recovery of the ovary to begin a new reproductive cycle. This pattern suggests that resumption of reproductive activity in Nile tilapia does not depend on complete prior elimination of all involutional structures, since this happens continuously in a way that is integrated with the development of new oocyte cohorts, as described in species that are important to ecology and aquaculture (Lowerre-Barbieri et al. 2011; Cassel et al. 2017b; Barbieri et al. 2018; Charitonidou et al. 2020).

The overall results of this study reinforce that ovarian regression in Nile tilapia constitutes a continuous, dynamic, and temporally organized process, characterized by alternation between mature follicles, atresia at different stages, and POCs. An integrated approach combining quantitative and histomorphological analyses of involutional structures is therefore essential for correct interpretation of the reproductive cycle in this species and also to provide relevant support for studies applied to reproductive management, aquaculture, and the conservation of natural stocks.

From the perspective of fish farming, our results fill an important gap regarding reproductive management of Nile tilapia (*O. niloticus*) broodstock. Although well-defined resting and reproductive reintroduction protocols exist for some farmed fish species, such as *Colossoma macropomum* (Streit Júnior et al. 2012), *Brycon opalinus* (Narahara et al. 2002), and *Piaractus mesopotamicus* (Bock and Padovani 2000), such guidelines have not yet been established for Nile tilapia, despite the widespread commercial use of this species. In general, interrupting contact with males is assumed to be an efficient strategy for promoting physiological recovery of females, contributing to the rapid resumption of reproductive activity (Castro et al. 2009; Yoshida et al. 2017). However, our findings demonstrate that the return to reproductive status is not immediate and depends on a minimum period for completion of the ovarian regressing processes and functional recovery of the ovary, including adequate reabsorption of follicular atresia and POCs.

In this context, our results consistently indicate that ovarian recovery in females is directly related to the duration of reproductive management, showing that prolonged maintenance in continuous reproduction favors the accumulation of degenerative structures, in turn compromising subsequent reproductive performance. Furthermore, our analysis demonstrates that a reproductive rest period of approximately 60 days is sufficient for the completion of involutional processes and for structural and functional reorganization of the ovary, resulting in adequate gonadal conditions to resume multiple-batch spawning. These results allow us to propose a reproductive management protocol for Nile tilapia fish farming in which the ovarian regression phase is recognized as a strategic stage of the production cycle. This protocol establishes a reproductive rest period of approximately 60 days, obtained through controlled unmating of females, an interval necessary for the completion of involutional processes as well as structural and functional recovery of the ovary. The females should only be reintroduced after this rest period is complete, ensuring adequate ovarian conditions to resume reproductive activity. This protocol offers evidence-backed potential to optimize the reproductive efficiency of broodstock, reduce losses associated with excessive atresia, and promote greater productive sustainability in tilapia farming systems.

## Conclusions

This study provides the first detailed temporal characterization of ovarian regressing phase in Nile tilapia, offering a more precise description of follicular atresia and POC dynamics and establishing a standardized histomorphological protocol for stage identification. Our findings show that the regressing phase is a critical transitional process rather than a functional pause, advancing reproductive biology but also offering practical applications for aquaculture in improved broodstock management and reproductive monitoring.

## Supplementary Information

Below is the link to the electronic supplementary material.

ESM 1 (DOCX 45.6 KB)

## Data Availability

Data is provided within the manuscript or supplementary information files.
