# Supplementary material for: Histomorphological and morphometric characterization of Nile tilapia (Oreochromis niloticus) ovaries, with emphasis on processes and structures related to the ovarian regressing phase
Source: Fish Physiol Biochem. 2026 Jul 10;52(4):116. doi: 10.1007/s10695-026-01740-x (PMC13354696; doi:10.1007/s10695-026-01740-x)
Supplement: Supplementary file 1 — (DOCX 45.6 KB) [file 10695_2026_1740_MOESM1_ESM.docx]

**Supplementary Table S1.** Results for statistical analysis of variance (Kruskal-Wallis test/H-statistic) and Wilcoxon paired test (W-statistic), with their respective P-values (p) for comparisons between the number of mature follicles and the number of atretic follicles (by involution stage) in the sampled groups. Significant results are highlighted in gray and are presented as an image in Figure 8.

|  | **Mature follicles** | **Initial atresia** | **Intermediate atresia** | **Advanced atresia** | **Final atresia** |
| --- | --- | --- | --- | --- | --- |
| **H** | 62.708 | 81.309 | 44.182 | 11.078 | 16.402 |
| **p** | 0.001 | 0.001 | 0.001 | 0.135 | 0.022 |

| **Mature follicles** | W_G0_ | p | W_G1_ | p | W_G2_ | p | W_G3_ | p | W_G4_ | p | W_G5_ | p | W_G6_ | p |
| --- | --- | --- | --- | --- | --- | --- | --- | --- | --- | --- | --- | --- | --- | --- |
| G0 |  |  |  |  |  |  |  |  |  |  |  |  |  |  |
| G1 | 2.609 | 0.009 |  |  |  |  |  |  |  |  |  |  |  |  |
| G2 | - | 0.976 | -2.744 | 0.006 |  |  |  |  |  |  |  |  |  |  |
| G3 | - | 0.617 | -2.670 | 0.008 | - | 0.913 |  |  |  |  |  |  |  |  |
| G4 | - | 0.449 | -2.074 | 0.038 | - | 0.163 | - | 0.139 |  |  |  |  |  |  |
| G5 | - | 0.443 | -3.736 | 0.001 | - | 0.860 | - | 0.704 | - | 0.061 |  |  |  |  |
| G6 | 2.671 | 0.008 | - | 0.880 | 3.594 | 0.001 | 3.759 | 0.001 | 2.340 | 0.019 | 3.745 | 0.001 |  |  |
| G7 | 4.162 | 0.001 | - | 0.145 | 4.571 | 0.001 | 4.667 | 0.001 | 3.557 | 0.001 | 4.978 | 0.001 | - | 0.193 |
|  |  |  |  |  |  |  |  |  |  |  |  |  |  |  |
| **Initial atresia** | W_G0_ | p | W_G1_ | p | W_G2_ | p | W_G3_ | p | W_G4_ | p | W_G5_ | p | W_G6_ | p |
| G0 |  |  |  |  |  |  |  |  |  |  |  |  |  |  |
| G1 | - | 0.102 |  |  |  |  |  |  |  |  |  |  |  |  |
| G2 | 1.956 | 0.047 | 2.130 | 0.034 |  |  |  |  |  |  |  |  |  |  |
| G3 | 3.086 | 0.002 | 3.368 | 0.001 | - | 0.083 |  |  |  |  |  |  |  |  |
| G4 | - | 0.995 | - | 0.447 | 1.970 | 0.046 | -2.753 | 0.006 |  |  |  |  |  |  |
| G5 | 2.600 | 0.009 | 3.395 | 0.001 | - | 0.075 | - | 0.827 | 2.651 | 0.008 |  |  |  |  |
| G6 | - | 0.181 | - | 0.830 | -2.506 | 0.012 | -3.716 | 0.001 | - | 0.081 | -3.376 | 0.001 |  |  |
| G7 | -4.315 | 0.001 | -3.061 | 0.002 | -4.927 | 0.001 | -5.643 | 0.001 | -3.768 | 0.001 | -5.817 | 0.001 | -2.613 | 0.009 |
|  |  |  |  |  |  |  |  |  |  |  |  |  |  |  |
| **Intermediate atresia** | W_G0_ | p | W_G1_ | p | W_G2_ | p | W_G3_ | p | W_G4_ | p | W_G5_ | p | W_G6_ | p |
| G0 |  |  |  |  |  |  |  |  |  |  |  |  |  |  |
| G1 | -2.734 | 0.006 |  |  |  |  |  |  |  |  |  |  |  |  |
| G2 | 2.912 | 0.004 | 5.067 | 0.001 |  |  |  |  |  |  |  |  |  |  |
| G3 | - | 0.167 | - | 0.314 | -3.616 | 0.001 |  |  |  |  |  |  |  |  |
| G4 | - | 0.748 | 2.217 | 0.027 | -2.928 | 0.003 | - | 0.296 |  |  |  |  |  |  |
| G5 | - | 0.463 | 2.257 | 0.024 | -3.542 | 0.001 | - | 0.181 | - | 0.666 |  |  |  |  |
| G6 | - | 0.069 | - | 0.230 | -3.700 | 0.001 | - | 0.990 | - | 0.165 | - | 0.180 |  |  |
| G7 | -2.592 | 0.010 | - | 0.702 | -4.435 | 0.001 | - | 0.172 | -2.330 | 0.020 | -2.303 | 0.021 | - | 0.106 |
|  |  |  |  |  |  |  |  |  |  |  |  |  |  |  |
| **Final atresia** | W_G0_ | p | W_G1_ | p | W_G2_ | p | W_G3_ | p | W_G4_ | p | W_G5_ | p | W_G6_ | p |
| G0 |  |  |  |  |  |  |  |  |  |  |  |  |  |  |
| G1 | - | 0.794 |  |  |  |  |  |  |  |  |  |  |  |  |
| G2 | - | 0.228 | 1.958 | 0.050 |  |  |  |  |  |  |  |  |  |  |
| G3 | - | 0.190 | - | 0.633 | -2.957 | 0.003 |  |  |  |  |  |  |  |  |
| G4 | - | 0.054 | - | 0.343 | -3.977 | 0.001 | - | 0.665 |  |  |  |  |  |  |
| G5 | - | 0.961 | - | 0.545 | - | 0.074 | -2.037 | 0.042 | 1.945 | 0.050 |  |  |  |  |
| G6 | - | 0.466 | - | 0.367 | - | 0.404 | 2.542 | 0.011 | 2.469 | 0.014 | - | 0.438 |  |  |
| G7 | - | 0.965 | - | 0.883 | - | 0.109 | - | 0.371 | - | 0.098 | - | 0.810 | - | 0.301 |

**Supplementary Table S2.** Results for statistical analysis of variance (Kruskal-Wallis test/H-statistic) and Wilcoxon paired test (W-statistic), with their respective P-values ​​(p) for comparisons between the number of mature follicles and the number of post-ovulatory complexes (POCs, by involution stage) in the sampled groups. Significant results are highlighted in gray and are presented as an image in Figure 9.

|  | **Mature follicles** | **Initial POC** | **Intermediate POC** | **Advanced POC** | **Final POC** |
| --- | --- | --- | --- | --- | --- |
| **H** | 62.708 | 40.466 | 37.126 | 30.437 | 47.207 |
| **p** | 0.001 | 0.001 | 0.001 | 0.001 | 0.001 |

| **Mature follicles** | W_G0_ | p | W_G1_ | p | W_G2_ | p | W_G3_ | p | W_G4_ | p | W_G5_ | p | W_G6_ | p |
| --- | --- | --- | --- | --- | --- | --- | --- | --- | --- | --- | --- | --- | --- | --- |
| G0 |  |  |  |  |  |  |  |  |  |  |  |  |  |  |
| G1 | 2.609 | 0.009 |  |  |  |  |  |  |  |  |  |  |  |  |
| G2 | 2.671 | 0.008 | - | 0.880 |  |  |  |  |  |  |  |  |  |  |
| G3 | 4.162 | 0.001 | - | 0.145 | - | 0.913 |  |  |  |  |  |  |  |  |
| G4 | 3.736 | 0.001 | - | 0.061 | - | 0.163 | - | 0.139 |  |  |  |  |  |  |
| G5 | 3.927 | 0.001 | - | 0.057 | - | 0.860 | - | 0.704 | -2.074 | 0.038 |  |  |  |  |
| G6 | - | 0.617 | 2.744 | 0.006 | 3.594 | 0.001 | 3.759 | 0.001 | 2.340 | 0.019 | 3.745 | 0.001 |  |  |
| G7 | - | 0.976 | 2.670 | 0.008 | 4.571 | 0.001 | 4.667 | 0.001 | 3.557 | 0.001 | 4.978 | 0.001 | - | 0.193 |
|  |  |  |  |  |  |  |  |  |  |  |  |  |  |  |
| **Initial POC** | W_G0_ | p | W_G1_ | p | W_G2_ | p | W_G3_ | p | W_G4_ | p | W_G5_ | p | W_G6_ | p |
| G0 |  |  |  |  |  |  |  |  |  |  |  |  |  |  |
| G1 | 3.753 | 0.001 |  |  |  |  |  |  |  |  |  |  |  |  |
| G2 | 3.537 | 0.001 | - | 0.637 |  |  |  |  |  |  |  |  |  |  |
| G3 | - | 0.198 | -2.530 | 0.011 | -2.272 | 0.023 |  |  |  |  |  |  |  |  |
| G4 | - | 0.061 | - | 0.460 | - | 0.195 | - | 0.202 |  |  |  |  |  |  |
| G5 | - | 0.171 | -2.316 | 0.021 | -2.458 | 0.014 | - | 0.712 | - | 0.075 |  |  |  |  |
| G6 | - | 0.138 | -2.218 | 0.027 | -2.391 | 0.017 | - | 0.974 | - | 0.118 | - | 0.897 |  |  |
| G7 | - | 0.607 | -2.943 | 0.003 | -3.250 | 0.001 | - | 0.232 | - | 0.082 | - | 0.201 | - | 0.181 |
|  |  |  |  |  |  |  |  |  |  |  |  |  |  |  |
| **Intermediate POC** | W_G0_ | p | W_G1_ | p | W_G2_ | p | W_G3_ | p | W_G4_ | p | W_G5_ | p | W_G6_ | p |
| G0 |  |  |  |  |  |  |  |  |  |  |  |  |  |  |
| G1 | 3.350 | 0.001 |  |  |  |  |  |  |  |  |  |  |  |  |
| G2 | 2.934 | 0.003 | - | 0.703 |  |  |  |  |  |  |  |  |  |  |
| G3 | - | 0.127 | - | 0.111 | - | 0.070 |  |  |  |  |  |  |  |  |
| G4 | 2.066 | 0.039 | -2.009 | 0.045 | -2.625 | 0.009 | - | 1.000 |  |  |  |  |  |  |
| G5 | - | 0.785 | -3.032 | 0.002 | -3.334 | 0.001 | - | 0.093 | -2.309 | 0.021 |  |  |  |  |
| G6 | 1.987 | 0.047 | -1.969 | 0.049 | -2.023 | 0.043 | - | 0.949 | - | 0.945 | 2.040 | 0.041 |  |  |
| G7 | - | 0.180 | -3.316 | 0.001 | -3.330 | 0.001 | - | 0.089 | -3.127 | 0.002 | - | 0.317 | -2.754 | 0.006 |
|  |  |  |  |  |  |  |  |  |  |  |  |  |  |  |
| **Advanced POC** | W_G0_ | p | W_G1_ | p | W_G2_ | p | W_G3_ | p | W_G4_ | p | W_G5_ | p | W_G6_ | p |
| G0 |  |  |  |  |  |  |  |  |  |  |  |  |  |  |
| G1 | - | 0.273 |  |  |  |  |  |  |  |  |  |  |  |  |
| G2 | - | 0.065 | - | 0.102 |  |  |  |  |  |  |  |  |  |  |
| G3 | - | 0.317 | - | 0.180 | -3.165 | 0.002 |  |  |  |  |  |  |  |  |
| G4 | - | 0.124 | - | 0.236 | - | 0.106 | 2.530 | 0.011 |  |  |  |  |  |  |
| G5 | - | 0.465 | - | 0.786 | - | 0.095 | - | 0.109 | - | 0.566 |  |  |  |  |
| G6 | - | 0.137 | - | 0.537 | - | 0.380 | 2.401 | 0.016 | - | 0.560 | - | 0.090 |  |  |
| G7 | - | 0.317 | - | 0.109 | -3.165 | 0.002 | - | 0.278 | -2.530 | 0.011 | - | 0.109 | -2.401 | 0.016 |
|  |  |  |  |  |  |  |  |  |  |  |  |  |  |  |
| **Final POC** | W_G0_ | p | W_G1_ | p | W_G2_ | p | W_G3_ | p | W_G4_ | p | W_G5_ | p | W_G6_ | p |
| G0 |  |  |  |  |  |  |  |  |  |  |  |  |  |  |
| G1 | 2.060 | 0.039 |  |  |  |  |  |  |  |  |  |  |  |  |
| G2 | 3.983 | 0.001 | - | 0.340 |  |  |  |  |  |  |  |  |  |  |
| G3 | 2.701 | 0.007 | - | 0.484 | - | 0.134 |  |  |  |  |  |  |  |  |
| G4 | 3.691 | 0.001 | - | 0.118 | - | 0.507 | - | 0.339 |  |  |  |  |  |  |
| G5 | 2.552 | 0.011 | - | 0.286 | - | 0.318 | - | 0.776 | - | 0.552 |  |  |  |  |
| G6 | 2.032 | 0.042 | - | 0.799 | -2.347 | 0.019 | - | 0.376 | -1.946 | 0.050 | - | 0.082 |  |  |
| G7 | - | 0.317 | 2.244 | 0.025 | -3.330 | 0.001 | -2.701 | 0.007 | -3.691 | 0.001 | -2.552 | 0.011 | -2.032 | 0.042 |
